# Concordance between GPS-based smartphone app for continuous location tracking and mother’s recall of care-seeking for child illness in India

**DOI:** 10.7189/jogh.08.020802

**Published:** 2018-12

**Authors:** Siddhivinayak Hirve, Andrew Marsh, Pallavi Lele, Uddhavi Chavan, Tathagata Bhattacharjee, Harish Nair, Harry Campbell, Sanjay Juvekar

**Affiliations:** 1 KEM Hospital Research Centre, Pune, India; 2 Institute for International Programs, Johns Hopkins University Bloomberg School of Public Health, Baltimore, Maryland, USA; 3 INDEPTH Network, East Legon, Accra, Ghana; 4 Usher Institute of Population Health Sciences and Informatics, University of Edinburgh, Edinburgh, Scotland, UK; * Joint first author with equal contributions; ** Joint last author with equal contributions

## Abstract

**Background:**

Traditionally, health care-seeking behaviour for child illness is assessed through population-based national demographic and health surveys. GPS-based technologies are increasingly used in human behavioural research including tracking human mobility and spatial behaviour. This paper assesses how well a care-seeking event to a health care facility for child illness, as recalled by the mother in a survey setting using questions sourced from Demographic and Health Surveys, concurs with one that is identified by TrackCare, a GPS-based location-aware smartphone application.

**Methods:**

Mothers residing in the Vadu HDSS area in Pune district, India having at least one young child were randomly assigned to receive a GPS-enabled smartphone with a pre-installed TrackCare app configured to record the device location data at one-minute intervals over a 6-month period. Spatio-temporal parameters were derived from the location data and used to detect a care-seeking event to any of the health care facilities in the area. Mothers were asked to recall a child illness and if, where and when care was sought, using a questionnaire during monthly visits over a 6-month period. Concordance between the mother’s recall and the TrackCare app to identify a care-seeking event was estimated according to percent positive agreement.

**Results:**

Mean concordance for a care-seeking event between the two methods (mother’s recall and TrackCare location data) ranged up to 45%, was significantly higher (*P*-value <0.001) for care-seeking at a hospital as compared to a clinic and for a health care facility in the private sector compared to that in the public sector. Overall, the proportion of disagreement for a care-seeking event not detected by TrackCare but reported by mother ranged up to 77% and was significantly higher (*P*-value <0.001) compared to those not reported by mother but detected by TrackCare.

**Conclusions:**

Given the uncertainty and limitations in use of continuous location tracking data in a field setting and the complexity of classifying human activity patterns, additional research is needed before continuous location tracking can serve as a gold standard substitute for other methods to determine health care-seeking behaviour. Future performance may be improved by incorporating other smartphone-based sensors, such as Wi-Fi and Bluetooth, to obtain more precise location estimates in areas where GPS signal is weakest.

Despite significant progress, acute diarrheal and respiratory infections continue to be the most common cause of under-five child mortality and morbidity in low and middle income countries [1]. Most deaths are preventable with an integrated strategy of preventive interventions (eg, by immunisation) coupled to timely access to appropriate health care services [2]. The world’s nations are mandated to achieve universal health coverage by 2030 as an essential step towards the sustainable development goals [3]. The WHO uses health seeking behaviour for child illness as one of the 16 essential health services to monitor progress towards the level and equity of universal health coverage in countries [4]. Health seeking behaviour determines utilization of health facilities which in turn aids planning national resources for disease control programs [5].

Traditionally, health care-seeking behaviour for child illness is assessed through population-based and nationally representative demographic and health surveys such as the National Family Health Survey (NFHS) in the Indian context. The primary caregiver (typically the mother) is asked to recall if the child had an illness (diarrhoea, fever, cough etc.) within the last two weeks, if and where treatment was sought and details of timeliness and appropriateness of treatment [6]. Maternal reports of care-seeking behaviour are subject to recall bias that varies by the ability to recall, severity of illness, period of recall and social desirability norms, and can significantly bias the estimate of actual behaviour [7]. Maternal recall of antibiotic treatment for pneumonia in children is a poor proxy indicator for pneumonia treatment rates [8]. On the other hand, maternal care-seeking reports has been shown to be a valid measure of care-seeking for child illness where utilization of public sector providers is high [9]. More research is needed to validate the accuracy of maternal reports of care-seeking for child illness in a survey setting.

Alternate methods including direct observation, participant diaries and health care facility records have been used to measure care-seeking behaviour, each with its own strength and limitation in terms of scope, effort, practicality, accuracy and potential for bias [10]. Technology-driven alternatives to measure care-seeking have been proposed to address some of these limitations. Early evidence from adapting survey questions on childhood illness to SMS-based approach has shown high agreement when comparing this approach to traditional, in-person survey administration [11], though such an approach remains dependent on active participant engagement. Alternately, sensors included within many mobile phones provide a platform for passively assessing participant health behaviour while minimizing participant burden [12].

Indoor location technologies such as radio frequency identification [13], Wi-Fi [14], GSM [15], Bluetooth® [16], infrared [17] or ultrasound [18] though accurate, are proximity dependent with their scope limited to only those fixed indoor locations where the receiver devices can be placed. In contrast, GPS-based technologies can be used to track mobility on a continuum as long as the satellite communication signal is unhindered and strong enough [19]. GPS technology has been used to track transportation [20], physical activity [21,22], human mobility [23,24], animal mobility and transmission of infectious diseases [25], monitoring of vaccination programs [26-29], hospitalization [30] and other areas of public health research [31]. The popularity, low cost, near-universal availability and widespread use of GPS-based location-aware technologies in smartphones provide an opportunity to track human mobility in time and space to a very fine scale [32]. As GPS technology evolves rapidly, there have been attempts to develop best-practice guidelines for the collection, transmission, processing and analysis of large quantities of location-based data so as to reduce data errors that are inherent to the GPS technology [33].

Maharashtra is the third largest state by area and second most populous with more than 110 million population (Census of India 2011). It is the wealthiest and most industrialized state and contributes to about 14% of India’s gross domestic product [34]. Mobile phone penetration has increased exponentially in India since 2004. It has a mobile phone subscriber base of more than 83 million accounting for about 8% of the total subscriptions in India in 2016 [35]. Ninety seven percent of urban and 86% of rural households in Maharashtra have a mobile phone. Sixty percent of urban and 31% of rural women possess a mobile phone that they themselves use [6]. The smartphone industry is a rapidly growing market with about a third of all mobile phone users in India expected to use smartphones by 2017, predominantly based on the Android operating system [36]. Mobile internet surfing habits show that more than 80% of the users are males less than 25 years of age. The most common use of smartphones is for browsing the internet (72%), following social media (56%), playing games (46%) and instant messaging (37%) [37].

GPS-enabled devices when properly deployed have been shown to have good spatial congruence between continuous GPS tracking and questionnaire data [38]. The extensive penetration of mobile connectivity in rural India provided an opportunity to use an Android-based geo-location mobile app (TrackCare) installed on the mother’s smartphone to track her visit to a health facility and validate it with her recall of a health facility visit [39]. The aim of this paper is to compare a mother’s report of care-seeking for a child illness during the 15 days prior to each follow-up visit with a care-seeking event as recorded by the TrackCare app installed on a GPS-enabled smartphone carried by the mother.

## METHODS

The CONSORT statement for randomized trials of non-pharmacologic treatments was used to guide the process and reporting of this study [40].

### Study area

**Figure 1:**
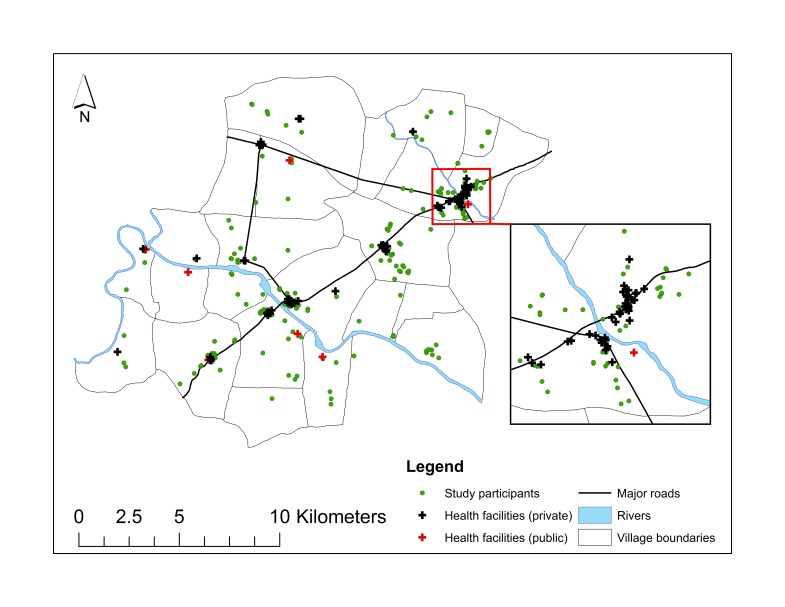
Map of study area showing location of HCF and study participants, Vadu, India. Map zoomed in the inset to show the high density of health care facilities in one of the villages of the study area.

The Improving Coverage Measurement for Maternal, Newborn and Child Health Study was conducted in 22 villages in Pune district in Maharashtra State in the western region of India. The study area comprises a population of about 143 000, is situated about 30 km from Pune city metropolis with rapid urbanization and industrialization [41]. Two rural hospitals, several health centres and more than a hundred private medical practitioners supported by 68 chemists provide medical care. The majority (71%) of these providers are situated in four villages along the main State highway that passes through the study area (**Figure 1**).

### Study design

**Figure 2:**
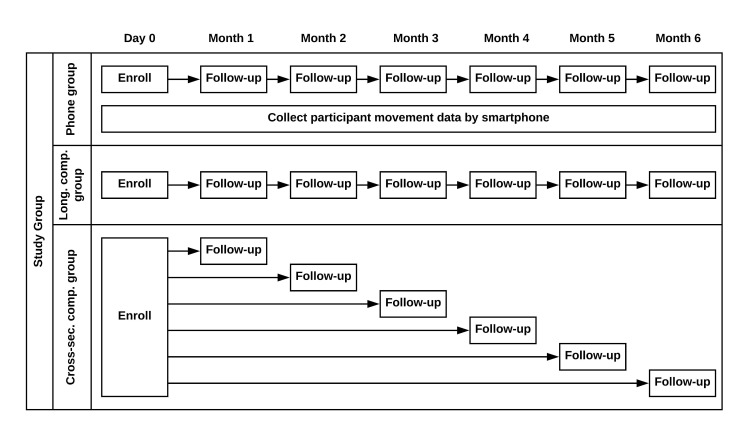
Participant follow-up schedule. Cross-sec. comp. group – Cross-sectional Comparison Group; Long. comp. group – Longitudinal comparison group.

A total of 926 mothers aged 15 – 49 years having at least one living child under the age of five years were randomly sampled from a population database maintained by the Vadu Health and Demographic Surveillance System. The mothers who were sampled were further randomly assigned to one of three groups - longitudinal phone group (200 mothers given a smartphone with a TrackCare App who were followed up monthly for 6 months), longitudinal control group (100 mothers who were followed up monthly for 6 months), and six cross-sectional control groups (about 75 mothers in each group who were followed up on one occasion during the six month period) (**Figure 2**). Field workers approached mothers at home for recruitment, 749 of whom (response rate 81%) consented to participate in the study. The investigators were not blinded to the mother’s group assignment. The study included a longitudinal control group to adjust for the potential bias in reporting care-seeking due to the presence of the study phone. The cross-sectional control groups were included to determine whether changes in care-seeking reports were due to repeated administration of the survey questionnaire. Sample size for the phone group was calculated based on an estimated 15-day care-seeking prevalence of 20% [42], an average of two eligible children per enrolled mother, a base concordance of accurate care-seeking of 80%, and a precision level of 8%.

### Survey data

Baseline information collected from mothers included individual and household demographic and socio-economic characteristics, and care-seeking preferences for child illness (Appendix S1 in **Online Supplementary Document(Online Supplementary Document)**). Mothers in the phone group were asked to rate their potential concerns (about damage, theft, loss of phone, personal safety, etc.) regarding the use of smartphone. These questions were developed based on our experiences with devices used for direct electronic data capture in the Vadu Health and Demographic Surveillance and other studies [43]. At each follow up, questions identical to those in the NFHS questionnaire were used to ask the mother to recall if the child had diarrhoea, fever or cough within the last 15 days, if care was sought, and the type of provider. Additional questions were asked to find when (how many days before the follow up) and where (name of health care facility) the care was sought. Mothers in phone group were further asked whether the study smartphone was carried during the visit to the health care provider. A separate health care facility survey identified and collected geo-coordinates of all fixed health care facilities (hospitals, clinics, health centres, chemist shops etc.) in the area.

### TrackCare mobile app

Mothers in the phone group received a dual SIM, GPS-enabled Sony Xperia E4 smartphone with one SIM card and the pre-installed TrackCare app. To encourage its use by the mother, all costs towards voice call, instant messaging and data usage was paid for by the study. Mothers could additionally opt to install their personal SIM card in the secondary SIM slot to ensure continuity in use of their existing SIM card and mobile number. Mothers were trained by the field investigator during home visit in the key features of the smartphone, instructed not to change the phone location access settings (set to high accuracy – ie, the location data sourced and retained from the better of the two sources viz. the network provider and the GPS chipset itself), ensure that the phone battery was kept charged during the whole day, and encouraged to carry and use the smartphone when moving outside of home. The smartphone settings were checked, any deviations from optimal settings were corrected, and the mother’s training was reinforced at each follow up visit. The TrackCare app did not require any user input and would run in the background when the smartphone was turned on, or automatically restart if forced to stop by the user. Moreover, a password prevented its accidental or intentional uninstallation by any participant. The TrackCare app was configured to record and save the device location data (latitude, longitude and positional accuracy), source of the location data (GPS chipset, mobile network), location mode (high accuracy, device only, battery saving option enabled, location services disabled) and the timestamp, at one-minute intervals throughout the 6-month follow up period. The TrackCare app was configured to upload the saved location data and the upload timestamp at hourly intervals (or in case of poor network signal, at the next scheduled interval), to a secured central study server database which in turn would synchronize real-time with another secured mirror image server. The location data was cleaned to remove duplicate records and cached data (arising when the TrackCare app used the previous location data to infer the present location in the case of a weak GPS or network signal strength) and incorrect timestamps were adjusted, so restricting the data set to a single coordinate for each minute. When multiple geo-coordinates were recorded during the same time interval, the geo-coordinate from the most robust location data source was retained. GPS data with low accuracy due to poor signal strength or environment interference were removed. Missing values were interpolated between geo-coordinates that were less than an hour apart or within 100 m of each other [39].

### Data analysis

The analysis for this paper was restricted to mothers from the phone group only, seeking care at qualified health care providers (practitioners of modern and Indian systems of medicine at hospitals, health centres and clinics) for their child’s illness. Care sought from Accredited Social Health Activists (ASHA), and Integrated Child Development Service (ICDS) workers was excluded as they typically provide peripatetic services once or twice a month and are accessed infrequently by mothers for curative care for child illness in the study setting. Pharmacies, drug-stores and shops were also excluded as infrequent secondary sources to access medicines following a visit to the health care provider. Furthermore, these sources often shared the same or adjoining location and are hence unsuited for separate detection from the health facility.

The location data transmitted by the study phones was used to identify a temporal and spatial clustering of geo-coordinates around any of the health care facilities indicative of a care-seeking event based on four parameters – (1) phone proximity to health care facility (proximity range), (2) minimum time period spent in proximity of health care facility (minimum time), (3) maximum time period spent in proximity of health care facility (maximum time), and (4) time spent outside proximity of health care facility within this minimum and maximum period (time outside) to account for the random error in the location data (jitter) due to the varying signal strength and positional accuracy. Proximity range, minimum time, and time outside are analogous to parameters used to identify visited locations from participant trajectory data [44]. Maximum duration was included to eliminate likely non-visits arising from participant’s whose daily movement brings them within range of a health care facility for extended periods of time. Furthermore, health care facilities situated near the mother’s home were excluded for that mother, as it would not be possible to differentiate between the clustering of the location data around the mother’s house and the health care facility. In general, a care-seeking event was defined if the mother’s phone localized within a certain proximity of a health care facility (excluding those which are situated near ie, within a certain distance of the mother’s home) for a minimum and maximum period of time, further allowing for a small continuous period of time, for the phone to localize outside the proximity of the health care facility within this period.

In the absence of a validated definition to identify a pattern of movement suspension or a trip to a health care facility based on GPS location data, we estimated the percent positive agreement for a care-seeking event (hereinafter referred to as concordance) ie, the agreement was the frequency with which the mother’s recall of a health facility visit matched with the location GPS data recorded by the TrackCare and defined as a potential visit using the parameters described above. We did not consider negative percent agreement (ie, agreement between the TrackCare and mother’s recall that a visit did not take place) in our concordance analysis as this would have falsely and highly inflated the overall concordance due to the large number of data points recorded by the TrackCare app outside the proximity of a health facility. Disagreement was also estimated between TrackCare and mother’s recall that a health facility visit did or did not take place. We did a sensitivity analysis using 6480 different threshold combinations for the various parameters to estimate concordance for the various parameters based on the GPS location data – proximity range (15 thresholds from 5 to 75 m with 5 m increments), minimum time (6 thresholds from 5 to 30 minutes with 5-minute increments), maximum time (6 thresholds of 2, 3, 6, 9, 12 and 24 hours), time outside (3 thresholds of 5, 10 and 15 minutes) and exclusion of health care facility if near mother’s home (4 thresholds of 50, 100, 200 m or no exclusion). We used multiple linear regression to model the effect of the parameters on concordance. The parameters with the different thresholds were treated as indicator variables to estimate the adjusted effect of each threshold of each parameter on the concordance between a mother’s recall and the TrackCare App for a care-seeking event. As a secondary objective, we also looked for concordance between TrackCare and mother’s recall of a care-seeking event specific to a calendar date. Within each level of the mother’s report, we analysed to see if the concordance varied by provider type (public or private), and type of health care facility (hospital or clinic).

### Ethical considerations

The study was approved by the Ethics Committee of the KEM Hospital Research Centre, Pune, India (Study No. 1415) and the University of Edinburgh, UK. Mothers provided written informed consent prior to enrolment and randomization. Prior to consent, mothers were informed that those assigned to the phone group would be allowed to keep the study phones even if they withdrew their participation at any stage of the study. Mothers in the phone group consented to the collection of their location data. The location data on the phone device was encrypted and erased as soon as it was transferred to the secured central study server.

The study was not registered with the Clinical Trials Registry as it was not considered by investigators to meet the criteria for such a trial. Study groups differed in the method and frequency with which their care-seeking behavior was measured but no group was provided with a health-related intervention intended to affect a health outcome.

## RESULTS

Of the 206 health care facilities identified during the health care facility census, 96 (47%) including 68 chemist shops were excluded from the analysis as many of them were located in the same premises as the hospital or clinic and it was not possible to differentiate between the two based on the GPS data. The remaining 110 facilities comprised of skilled providers in hospital (52%) and outpatient clinic (48%) settings, mostly in the private sector (94%). The majority of these providers (71%) were located in 4 larger villages (more than 7 health care facilities in each village) situated along the State highway compared to the remaining 18 smaller villages (**Figure 1**).

Two hundred mothers having a total of 324 children under five years of age were enrolled in the phone group between June and August 2015 (**Table 1**). One mother having one child withdrew consent prior to the first follow-up visit and was excluded from the analysis. More than 90% of the expected study visits were completed over the 6-month follow up period. The mean age of mothers was 25.3 years with a mean 10.9 years of schooling. About 54% lived in joint families, 42% had one living child, and 28% were currently employed. Sixty percent of all mothers came from the 4 larger villages whereas 40% resided in the other 18 villages. A significantly higher proportion (80%) of mothers from the larger villages were housewives compared to about 60% of mothers from the smaller villages. The proportion of mothers living in joint families was significantly lower in the larger villages. A significantly higher proportion of mothers from the larger villages (27.4%) were from the poorest quintile compared to 9.8% mothers from the smaller villages. A total of 413 child illness events were reported by mothers based on a 15-day recall over the 6-month follow up period. Mothers reported care-seeking for about 82% of these illness episodes, most frequently from a skilled provider (95%) based in a hospital facility (85%) setting in the private sector (93%).

**Table 1:** Participant and contextual characteristics, and child-illness seeking behaviour of phone group mothers (n = 199)

	Villages with low (≤7) HCF density	Villages with high (>7) HCF density	Overall
No. of mothers enrolled	82	117	199
Mother’s age (years) – mean (SD)	25.5 (3.5)	25.2 y (3.1)	25.3 y (3.3)
Mother’s education (years) – mean (SD)	10.6 (2.6)	11.1 y (2.7)	10.9 y (2.7)
Father’s education (years) – mean (SD)	10.6 (2.5)	11.9 y** (2.9)	11.4 y (2.8)
Mothers currently employed (%)	39.2%**	20.0%	28.0%
Mothers in joint family (%)	69.1%***	43.4%	54.1%
Under-five children in family (%):			
-One child	39.0%	44.4%	42.2%
-Two or more	61.0%	55.6%	57.8%
Distribution of wealth index quintiles:			
-Poorest	9.8%	27.4%***	20.1%
-2^nd^ quintile	15.9%	23.1%	20.1%
-3^rd^ quintile	17.1%	22.2%	20.1%
-4^th^ quintile	25.6%	16.2%	20.1%
-Richest	31.7%	11.1%	19.6%
No. of children enrolled	136	187	33
Child’s age (years):			
0 to <1	11.0%	7.5%	9.0%
1 to <2	19.9%	20.9%	20.4%
2 to <3	25.0%	17.6%	20.7%
3 to <4	16.2%	21.4%	19.2%
4 to <5	27.9%	32.6%	30.7%
No. of provider facility and type	58	148	206
-Skilled provider facility:‡	32 (55.2%)	78 (52.7%)	110 (53.4%)
-Public sector	15.6%	2.6%	6.4%
-Private sector	84.4%	97.4%	93.6%
-Inpatient or outpatient care	37.5%	57.7%	51.8%
-Outpatient care only	62.5%	42.3%	48.2%
Unskilled provider facility:†	26 (44.8%)	70 (47.3%)	96 (46.6%)
-Drug store	30.8%	85.7%	70.8%
-Anganwadi worker, ASHA	53.9%	14.3%	25.0%
-Other	15.4%	0%	4.2%
No. and prop. of child illness episodes reported by mother with a 15-day recall	170 (25.6%)	243 (26.3%)	413 (26.0%)
No. and prop. of care-seeking events for child illness reported by mother	129 (86.0%)	183 (78.9%)	312 (81.7%)
Care-seeking at skilled provider facility:†,‡	125 (98.4%)*	169 (92.9%)	294 (95.1%)
-Public sector	8.0%	10.1%	9.2%
-Private sector	92.0%	92.9%	92.5%
-Hospital (outpatient or inpatient care)	79.2%	89.9%	85.4%
-Clinic (outpatient care only)	22.4%	11.8%	16.3%
Care-seeking at unskilled provider facility§	5 (3.9%)	14 (7.7%)	19 (6.1%)

HCF – health care facility; SD – standard deviation; y – years, ASHA – Accredited Social Health Activist

*P-value <0.05; **P-value <0.01; ***P-value <0.001.

†Skilled provider facility includes hospital, health centre, clinic, practitioner of modern medicine and Indian systems of medicine.

‡Percentage sums for care-seeking from skilled and unskilled providers, public and private sector providers, and hospitals and clinics may exceed 100 due to multiple responses (eg, care-seeking from public and private sector providers during the same episode).

§Unskilled provider includes chemist shop, ICDS centre, traditional healer, friend and relative.

About 38.5 million location data points were uploaded by the TrackCare App. The shortfall in data points could be due to the phone being in a power-off mode or due to a discharged battery. After removing location data points with poor positional accuracy and outliers (about 1 million), about 6 million location data points were interpolated to replace the cached data and missing data, to yield a total of about 43.5 million location data points (84%, equivalent to 152 days distributed over the expected 180 observation days). Seventy-nine percent of mothers met all compliance criteria (phone available for inspection at visit, locations services set to high accuracy and optimally configured) for the correct use of smartphone during the follow up visits.

**Figure 3:**
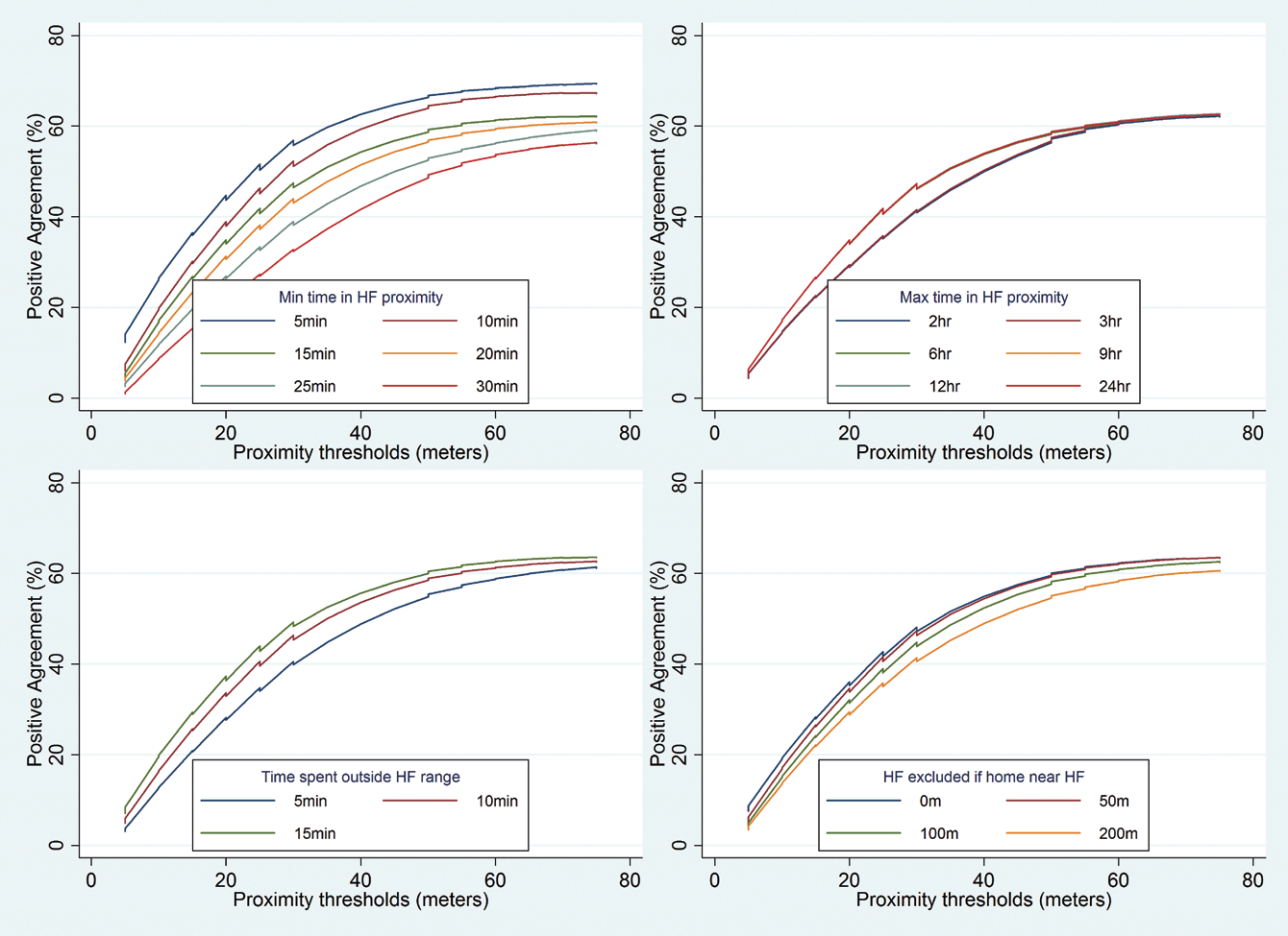
Effect of varying thresholds of different parameters on concordance between any care-seeking event defined by TrackCare app and mother’s recall. m – meters; min – minutes; hr – hours; HF – health facility.

**Figure 4:**
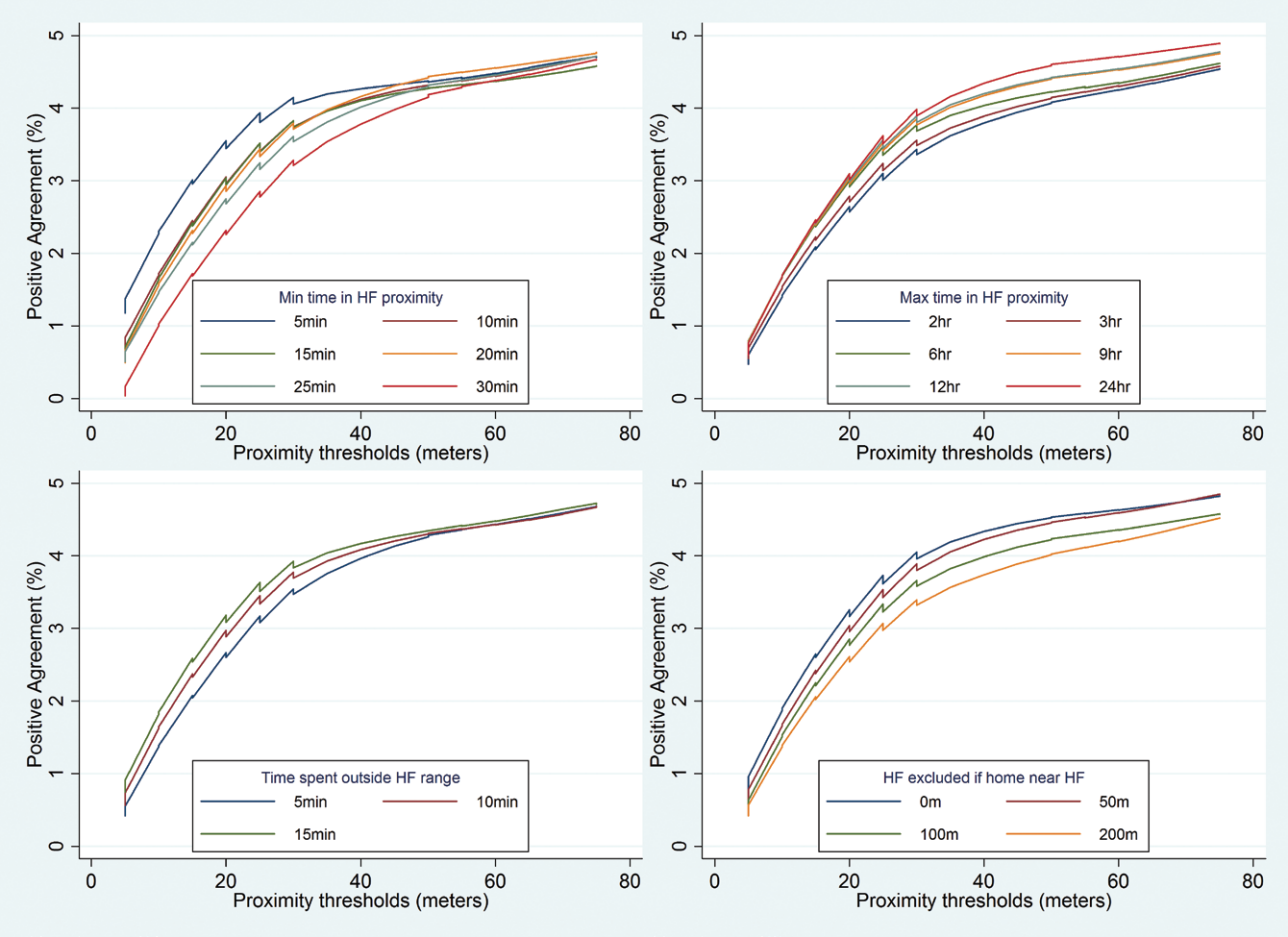
Effect of varying thresholds of different parameters on concordance between a date-specific care-seeking event defined by TrackCare app and mother’s recall. m – meters; min – minutes; hr – hours; HF – health facility.

The effect of varying the thresholds of different parameters on concordance between mother’s recall and the TrackCare location data to identify a care-seeking event for a child illness is shown in **Figure 3**. The mean concordance for a care-seeking event increased from 2.6% to 41.6% as the proximity range threshold increased from 5 to 75 m. The increase in concordance was maximal at the 15 m proximity threshold – with marginal increases thereafter (**Table 2**). Mean concordance decreased from 39% to 25.1% as the minimum time threshold increased from 5 to 30 minutes. However, the decrease was lower at a minimum time threshold of 15 minutes or more. The concordance varied marginally (about 1.6% and 4.7%) when the maximum time threshold varied between 2 to 24 hours or when the time outside threshold varied between 5 and 15 minutes respectively. Concordance decreased marginally from 32.8% to 29.5% when the health care facility near home threshold increased for excluding health care facilities that were near the mother’s home. The mean concordance between mother’s recall and the TrackCare location data to identify a ‘date-specific’ care-seeking event ranged between 0.1% to 3.8% and was significantly lower (*P*-value <0.001) than the concordance for any care-seeking event (**Table 2****,** **Figure 4**). Concordance between the mother’s recall and the TrackCare location data for a date-specific care-seeking event did not change significantly when the thresholds for proximity range, minimum time, maximum time, time outside, and health care facility near home, were varied (**Table 3**).

**Table 2:** Concordance between the TrackCare location data and mother’s recall for any care-seeking event or a date-specific care-seeking event

	Any care-seeking event in last 15 days			Date specific care-seeking event		
**Parameter threshold**	**% positive agree (SD)**	**Disagree – Visit not recalled by mother (SD)**	**Disagree –Visit not detected by TrackCare (SD)**	**% positive agree (SD)**	**Disagree – Visit not recalled by mother (SD)**	**Disagree –Visit not detected by TrackCare (SD)**
**Phone proximity to HCF (m):**						
5	2.6 (3.51)	3.6 (7.74)	93.9 (9.59)	0.1 (0.36)	6.2 (9.5)	93.6 (9.6)
10	11.3 (8.9)	10.9 (14.5)	77.8 (15.6)	1.2 (1.2)	23.6 (16.2)	75.1 (16.6)
15	20.1 (13.4)	16.7 (18.8)	63.2 (18.3)	2.0 (1.7)	40.4 (19.2)	57.6 (19.5)
20	24.8 (15.5)	20.9 (21.3)	54.3 (18.5)	2.2 (1.8)	49.3 (18.4)	48.5 (18.6)
25	28.9 (17.0)	23.6 (22.6)	47.5 (18.5)	2.3 (1.8)	56.7 (17.7)	40.9 (17.9)
30	31.4 (17.7)	25.4 (23.5)	43.2 (18.7)	2.6 (1.9)	60.5 (17.5)	36.9 (17.7)
35	33.3 (18.7)	27.8 (23.8)	38.8 (18.2)	2.9 (2.2)	64.1 (17.3)	33.0 (17.1)
40	37.1 (20.5)	30.9 (24.0)	32.0 (16.9)	3.1 (2.3)	68.6 (16.0)	28.4 (15.8)
45	38.7 (20.9)	32.1 (24.3)	29.2 (16.1)	3.1 (2.2)	71.1 (15.7)	25.8 (15.4)
50	39.2 (21.2)	33.1 (24.6)	27.7 (15.5)	3.1 (2.1)	72.9 (15.4)	24.1 (15.1)
55	40.4 (21.7)	34.0 (25.0)	25.5 (15.2)	3.1 (2.1)	74.1 (15.1)	22.7 (14.5)
60	40.9 (22.3)	35.5 (25.5)	23.6 (14.0)	3.2 (2.0)	76.1 (13.4)	20.7 (12.8)
65	41.3 (22.2)	36.1 (25.3)	22.7 (13.6)	3.2 (1.9)	77.3 (12.3)	19.5 (11.7)
70	41.6 (22.2)	36.4 (25.4)	22.0 (13.6)	3.5 (2.0)	78.4 (11.8)	18.1 (10.7)
75	41.6 (22.2)	36.9 (25.5)	21.5 (13.6)	3.5 (2.0)	79.5 (10.9)	17.0 (9.8)
**Minimum time spent in proximity of HCF (min):**						
5	39.0 (23.0)	32.4 (25.2)	28.6 (23.4)	3.1 (2.4)	70.0 (22.3)	26.9 (22.6)
10	35.1 (23.4)	30.0 (25.3)	34.9 (25.7)	2.5 (2.0)	65.1 (24.5)	32.4 (25.1)
15	32.3 (21.7)	27.7 (24.8)	40.0 (25.3)	2.6 (2.0)	61.5 (24.9)	35.9 (25.5)
20	30.1 (21.1)	26.1 (24.1)	43.8 (25.7)	2.6 (2.1)	58.4 (25.3)	39.0 (26.0)
25	27.6 (20.1)	24.3 (23.8)	48.1 (25.8)	2.5 (2.1)	54.7 (26.4)	42.8 (27.0)
30	25.1 (19.4)	21.2 (23.0)	53.7 (26.2)	2.4 (2.0)	49.9 (27.0)	47.7 (27.7)
**Maximum time spent in proximity of HCF (hr):**						
2	30.5 (21.4)	25.8 (24.6)	43.8 (27.0)	2.4 (2.0)	58.5 (26.3)	39.1 (27.0)
3	30.6 (21.5)	25.9 (24.7)	43.5 (27.1)	2.5 (2.0)	58.8 (26.3)	38.7 (26.9)
6	32.0 (22.2)	27.4 (24.6)	40.6 (26.4)	2.6 (2.1)	60.4 (25.7)	37.0 (26.3)
9	32.1 (22.3)	27.5 (24.7)	40.5 (26.5)	2.7 (2.1)	60.5 (25.7)	36.8 (26.4)
12	32.1 (22.3)	27.5 (24.7)	40.5 (26.5)	2.7 (2.1)	60.6 (25.8)	36.7 (26.4)
24	32.1 (22.2)	27.5 (24.6)	40.4 (26.5)	2.7 (2.2)	60.7 (25.8)	36.6 (26.5)
**Time spent outside proximity of HCF within minimum and maximum time (min):**						
5	29.0 (24.6)	25.0 (24.2)	46.0 (27.7)	2.4 (2.1)	56.3 (27.0)	41.3 (27.7)
10	31.9 (22.1)	27.2 (24.7)	40.9 (26.4)	2.6 (2.1)	60.6 (25.7)	36.8 (26.3)
15	33.7 (22.0)	28.6 (25.0)	37.7 (25.3)	2.8 (2.1)	62.9 (24.8)	34.3 (25.3)
**HCF near mother’s home (m):**						
no exclusion	32.8 (22.3)	28.5 (25.1)	38.7 (26.0)	2.8 (2.1)	64.8 (24.9)	32.5 (25.5)
50	32.6 (22.3)	27.6 (24.9)	39.8 (26.7)	2.7 (2.1)	62.1 (26.1)	35.3 (26.8)
100	31.3 (21.7)	27.0 (24.8)	41.8 (27.1)	2.6 (2.1)	58.9 (26.6)	38.6 (27.2)
200	29.5 (21.5)	24.6 (23.7)	45.9 (26.4)	2.4 (2.1)	54.0 (24.9)	43.5 (25.5)
**Type of HCF:**						
Hospital	40.2 (17.2)	15.7 (8.5)	44.1 (25.5)	3.7 (1.4)	56.6 (23.2)	39.8 (24.4)
Clinic	12.2 (6.5)	62.0 (24.0)	25.8 (25.7)	0.6 (0.5)	74.6 (25.6)	24.8 (25.8)
**HCF sector:**						
Public sector	12.6 (10.7)	33.3 (20.6)	54.1 (23.6)	1.3 (2.5)	50.6 (28.3)	48.1 (28.7)
Private sector	45.5 (18.4)	12.9 (8.1)	41.6 (25.4)	3.8 (1.4)	59.1 (23.0)	37.1 (24.3)
**Mother resides in:**						
high HCF density village	32.7 (22.5)	28.9 (26.3)	38.5 (28.5)	3.1 (2.3)	61.7 (26.7)	35.2 (27.5)
low HCF density village	30.4 (21.4)	25.0 (22.7)	44.6 (24.3)	2.1 (1.7)	58.1 (25.0)	39.7 (25.4)

m – meters; min – minutes; hr – hours; HCF – health care facility, SD – standard deviation; HCF – health care facility

**Table 3:** Modelling effect of varying the parameter thresholds on concordance of identifying care-seeking events based on GPS data and on mother’s recall

	Any care-seeking event		Date specific care-seeking event	
**Parameter**	β*	**(95% CI)**	β*	**(95% CI)**
**Phone proximity to HCF (m):**				
5	reference		reference	
10	7.5	(7.0–8.1)	0.9	(0.8–1.0)
15	16.2	(15.7–16.8)	1.7	(1.6–1.8)
20	20.7	(20.2–21.3)	1.8	(1.7–1.9)
25	24.8	(24.2–25.3)	1.9	(1.8–2.0)
30	27.4	(26.8–27.9)	2.2	(2.1–2.3)
35	29.2	(28.6–29.7)	2.7	(2.6–2.8)
40	32.5	(31.9–33.0)	2.9	(2.8–3.0)
45	34.0	(33.5–34.6)	3.0	(2.9–3.1)
50	34.2	(33.6–34.7)	2.9	(2.8–3.0)
55	35.2	(34.6–35.7)	3.1	(3.0–3.2)
60	35.2	(34.7–35.8)	3.1	(3.0–3.2)
65	35.8	(35.3–36.4)	3.0	(2.9–3.1)
70	35.9	(35.4–36.5)	3.8	(3.7–3.9)
75	36.0	(35.4–36.5)	3.7	(3.6–3.8)
**Minimum time spent in proximity of HCF (min):**				
5	reference		reference	
10	-5.4	(-5.8–-5.1)	-1.1	(-1.1–-1.0)
15	-7.3	(-7.6–-6.9)	-1.0	(-1.1–-0.9)
20	-10.3	(-10.7–-10.0)	-1.1	(-1.1–-1.0)
25	-12.9	(-13.3–-12.6)	-1.0	(-1.1–-1.0)
30	-14.9	(-15.2–-14.5)	-1.2	(-1.2–-1.1)
**Maximum time spent in proximity of HCF (hr):**				
2	reference		reference	
3	-0.0	(-0.4–0.3)	0.1	(-0–0.1)
6	1.0	(0.7–1.3)	0.2	(0.1–0.2)
9	1.1	(0.7–1.4)	0.2	(0.1–0.3)
12	1.1	(0.7–1.4)	0.2	(0.1–0.3)
24	1.1	(0.7–1.4)	0.3	(0.2–0.3)
**Time spent outside proximity of HCF within minimum and maximum time (min):**				
5	reference		reference	
10	3.2	(3.0–3.5)	0.4	(0.3–0.4)
15	5.3	(5.1–5.5)	0.7	(0.7–0.8)
**HCF near mother’s home (m):**				
0	reference		reference	
50	-0.3	(-0.6–-0.0)	-0.1	(-0.1–-0.0)
100	-1.4	(-1.7–-1.1)	-0.2	(-0.3–-0.2)
200	-3.2	(-3.5–-2.9)	-0.3	(-0.4–-0.3)
**Type of HCF:**				
Clinic	reference		reference	
Hospital	28.0	(27.8–28.2)	3.13	(3.11–3.14)
**HCF sector:**				
Private	reference		reference	
Public	-34.9	(-33.1–-32.7)	-2.4	(-2.5–-2.4)
**Mother resides in:**				
High HCF density village	reference		reference	
Low HCF density village	-6.2	(-6.4–-6.0)	-1.6	(-1.7–-1.6)

CI – confidence interval; m – meters; min – minutes; hr – hours; HCF – health care facility

*β indicates the percent increase or decrease in concordance compared to the reference category of the parameter threshold.

Overall, the proportion of care-seeking events not detected by TrackCare but reported by mother was higher compared to the proportion of care-seeking events not reported by mother but detected by TrackCare, for all combinations of parameters and thresholds. This disagreement (TrackCare does not detect) was high with a more restrictive definition of a care-seeking event and decreased as the definition became permissive.

**Figure 5:**
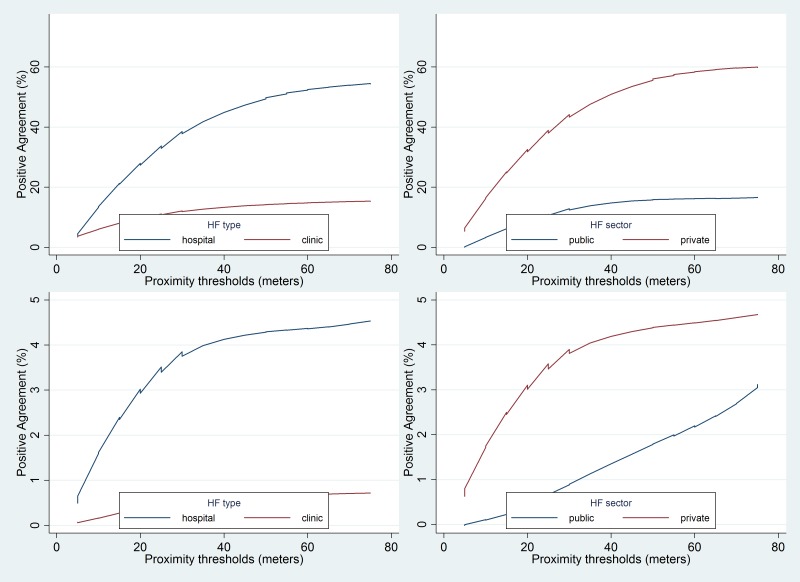
Concordance for any care-seeking event (top panel) and date-specific care-seeking event (bottom panel) defined by TrackCare app and mother’s recall by type of health care facility (the y-axis scale is different for the top and bottom panels). HF – health facility

The mean concordance between mother’s recall and TrackCare location data was significantly higher for a care-seeking event at a hospital (40.2%) as compared to a care-seeking event at a clinic (12.2%). Similarly, the mean concordance was significantly higher for care-seeking in the private sector (45.5%) as compared to care-seeking in the public sector (12.6%) (**Table 2****,** **Table 3****,** **Figure 5**). Concordance was similar (30.4% and 32.7%) for mothers residing in villages with low or high density of health care facilities respectively.

## DISCUSSION

GPS-based technologies are increasingly used in human behavioural research including tracking human mobility and spatial behaviour [45,46]. There is an immense potential to couple GPS technologies along with other sensors such as accelerometers, air pollution sensors etc. to study human spatial behaviour and disease dynamics [47]. Our study was originally designed to validate a mother’s recall of a care-seeking event for child illness using identical questions from the NFHS against a presumable gold standard viz. a health facility visit identified by a GPS-based location aware smartphone technology. Given the complexity of defining a temporary suspension of mobility at a health facility with continuous location tracking data, we realized that the location data would not serve as a gold standard for validation and we changed our analytic approach from validation to that of concordance.

Mean concordance on a care-seeking event between the two methods ranged up to 45%, was significantly higher for care-seeking at a hospital as compared to a clinic and for a health care facility in the private sector compared to that in the public sector. In contrast, concordance was significantly lower (ranged up to 3.8%) for a date-specific care-seeking event. This could be partly because the care-seeking event date was an approximation derived by subtracting the mother’s recall of the number of days ago that the care-seeking event took place from the date of interview. Further, even if the mother had been asked to recall the exact care-seeking event date, the concordance for the date-specific event would still likely be low as the mother’s recall of the exact event date would be also prone to error. Disagreement was mostly due to care-seeking events that were reported by the mother but missed by TrackCare among more restrictive GPS-based definitions of a care-seeking event. As the thresholds used to define a care-seeking event became more permissive, the source of disagreement switched to visits detected by TrackCare but not reported by the mother. A restrictive definition would require a mother to be nearer to a health facility to qualify as a visit (eg, 5 m) while a permissive definition would allow a higher threshold (eg, 75 m) for what qualifies as a visit. Visits that were not detected by TrackCare even with the most permissive thresholds may indicate a possible failure of the GPS-based method due to a switched-off phone, or on the other hand when a mother falsely reports a health facility visit because of a social desirability bias.

Given the accuracy, quantum and high resolution of location-based data, it is tempting to validate classic survey instruments with the ‘gold standard’ GPS data [48]. GPS provides highly accurate information to track routes taken to places visited under perfect weather and satellite geometry conditions. However in real-life field conditions, GPS signals are prone to error due to sub-optimal weather conditions, signal obstruction, signal noise, battery life, and poor compliance of use that results in mobility or (suspension of mobility) estimates that are uncertain [44]. Nevertheless, efforts have been made to establish methodological and analytic standards to accurately measure endpoints such as patterns of suspension of movement or spatio-temporal clustering of location data that may in turn define a stop or a visit to a place of interest [49,50]. Further, guidance on how many and which temporal and spatial parameters and their optimal thresholds are needed to best define a temporary suspension or stop or a visit to the place of interest is context-specific as signal strength, accuracy of the location data will vary across time and place. In our study, TrackCare was configured to record GPS data at one-minute intervals as increasing the frequency of GPS data collection does not translate to an increase in concordance to capture mobility based on interviews and GPS data [44].

Our study faced several methodological and analytic challenges. First, processing the large quantum of raw GPS data into potential and meaningful location visits was a major challenge. We used a simple approach based on clustering of coordinates in time and space to detect a potential visit to a health care facility. Second, in less than perfect field conditions, the GPS data was prone to several types of errors due to poor signal strength, signal obstruction, battery life which may force a change to a less robust source for the location data. Various signal processing algorithms have been proposed to reduce such errors and improve detection of activity location [51-54]. Our study used a linear interpolation to impute coordinates for missing values that were less than 1 hour apart or within 100 m of each other. Third, our computing hardware and software capacity was suboptimal to process the large volume of location data and it took several days to complete just one run of some of the statistical scripts that were written for the sensitivity analysis of the 6480 threshold combinations for identifying a care-seeking event. These challenges could have been addressed with access to cloud computing services and more efficient statistical scripts which would allow segmenting of data to cut down on processing time. Fourth, issues of battery life, compliance in the correct use of the smartphone, and use by other household members had the potential to compromise the completeness and quality of data, as evidenced in a systematic review of 24 studies that used GPS to study physical activity and the environment [22]. Our study succeeded in collecting 84% of the expected GPS data points over the 6-month follow up period. About 5% of the GPS data points were excluded due to low positional accuracy of more than 50 m [39]. Fifth, the exclusion of chemist shops as health care facilities from our analysis could potentially cause some misclassification error as in the case where the mother visited the chemist shop directly for medicines without a doctor’s consultation, but the GPS data classified it as a visit to the hospital or clinic in the immediate proximity. Even so, we believe that such an error would be small. Sixth, we included about 10 health care facilities from outside the study area that mothers commonly reported as preferred health care providers for child illness. Mothers who reported to have sought care at facilities outside the study area other than those included in the study, would be missed by the TrackCare App. However, the number of care-seeking events at these other (non-included) health care facilities outside the study area was small and would have marginally increased the discordance between the two methods. Seventh, we excluded the percent agreement for a non-event when we estimated concordance. It was intuitive to do so as the main purpose was to identify a care-seeking event (rather than a non-event). Moreover, the high levels of agreement for a non-event, if included, would have masked the agreement for a care-seeking event. Lastly, GPS-defined health facility visit assumes the purpose of seeking health care and are not able to differentiate between hospital visits for care-seeking from that for other reasons such as employment, visiting patients admitted at the health facility.

The low levels of concordance seen between the GPS-based method and the self-report by a mother, for a care-seeking event for child illness could be due to technology-related limitations, an imperfect capture of health care facility visits by the parameters and thresholds, or a mother’s failure to recall. More research is needed to develop algorithms that use continuous location tracking data to better define a temporary suspension of movement or a visit to a place of interest.The identification of an approach, including which parameters to include and their optimal values, should be conducted with consideration of the local context and research objectives. Wherever gold standard mobility information are available, these may be compared against the inferences drawn from an approach to optimize its performance. These approaches may also incorporate additional smartphone sensors, such as Wi-Fi and Bluetooth, to more precisely determine participant location, especially indoors where GPS signal is weakest [12,55]. The use of TrackCare and other similar GPS-based apps on smartphones to better understand health seeking-care pathways, provider shopping, provider preference, referral and bypass patterns for acute and chronic health conditions, needs to be explored. The use of such novel tools needs to consider local and cultural concerns of the population. More research is needed to compare with other sophisticated approaches based on artificial intelligence to reduce the uncertainty in detecting locations visited by participants based on GPS data [56].

## CONCLUSION

Given the uncertainty and limitations in use of continuous location tracking data in a field setting and the complexity of classifying human activity patterns, continuous location tracking may not serve as a gold standard substitute for other methods to determine health care-seeking behaviour. Even as we better understand the processing and interpretation of continuous location tracking data, GPS-based tools are useful adjuncts but not yet substitutes to the classical survey instruments, to help understand the bias and validate responses related to activity visits such as care-seeking behaviour.
