# Supplementary material for: Concordance between GPS-based smartphone app for continuous location tracking and mother’s recall of care-seeking for child illness in India
Source: J Glob Health. 2018 Sep 19;8(2):020802. doi: 10.7189/jogh.08.020802 (PMC6209739; doi:10.7189/jogh.08.020802)
Supplement: Online Supplementary Document [file jogh-08-020802-s001.pdf]

Online Supplementary Document

Hirve et al. Concordance between GPS-based smartphone app for continuous location tracking and mother's recall of care-seeking for child illness in India

J Glob Health 2018;8:020802

Investigating Validity of Maternal Recall of Care-seeking Location

Collaborators: *Vadu Health and Demographic Surveillance System, Vadu Rural Health Program, KEM Hospital Research Centre Pune and University of Edinburgh, United Kingdom*

Form 1: Baseline questionnaire

(to be administered at enrollment for all mothers)

Starting time of the interview: □□:□□			Date: □□/□□/□□□□	
Interviewer Name:			Mother's Name:	
HDSS Id :□□□ / □□□ / □□□□ Village Wadi Household Number				
Study ID Number: □□□	Study Group ID: □ (See codes below)		Phone ID Number: □□□ (Enter 998 if N/A)	
(1)	GPS Device ID □□	(2)	Number of satellites in range	□□
(3)	Accuracy □□	(4)	Elevation	□□□
(5)	Latitude	North N	Degree and decimal □□.□□□□□□	
(6)	Longitude	East E	Degree and decimal □□.□□□□□□	
(7)	Way Point	01 In front of household	02 Place near household:	

Codes for Study Group ID:

Phone Group	1
Longitudinal comparison group (visit at each follow-up)	2
Cross-sectional comparison group 1 (visit once at follow-up 1)	3
Cross-sectional comparison group 2 (visit once at follow-up 2)	4
Cross-sectional comparison group 3 (visit once at follow-up 3)	5
Cross-sectional comparison group 4 (visit once at follow-up 4)	6
Cross-sectional comparison group 5 (visit once at follow-up 5)	7
Cross-sectional comparison group 6 (visit once at follow-up 6)	8

No.	Questions and Filters	Coding Categories	SKIP
(8)	How old were you at your last birthday?	Age in completed years _ _	
(9)	Have you ever attended school?	Yes 1<br>No 2	 →11
(10)	What was the highest standard or degree / diploma you completed?<br><br>RECORD THE RESPONSE AS THE NUMBER OF YEARS OF EDUCATION COMPLETED	Completed years of education _ _	

(11)	Are you currently employed?	Yes<br>No	1<br>2	→13
(12)	Have you been employed for at least a total period of 3 months in the previous 12 months?	Yes<br>No	1<br>2	 →14
(13)	What is your principal occupation, that is, what kind of work do you mainly do?<br><br>RECORD ONLY ONE OPTION. IF A RESPONDENT REPORTS MULTIPLE OCCUPATIONS, RECORD THE OCCUPATION WHERE SHE SPENDS RELATIVELY MORE WORKING TIME.	Cultivators<br>Agricultural laborer<br>Worker in household industry<br>Other worker<br>Student<br>Household duties<br>Dependent<br>Pensioner<br>Rentier<br>Beggar<br>Other ______________________<br>(Specify)<br>No answer/do not want to answer	01<br>02<br>03<br>04<br>05<br>06<br>07<br>08<br>09<br>10<br>11<br><br>99	
(14)	Do you own this house or any other house either alone or jointly with someone else?	Alone only<br>Jointly only<br>Both alone and jointly<br>Does not own	1<br>2<br>3<br>4	
(15)	Do you own any land either alone or jointly with someone else?	Alone only<br>Jointly only<br>Both alone and jointly<br>Does not own	1<br>2<br>3<br>4	
(16)	What is your current marital status?	Currently married<br>Married, gauna not performed<br>Widowed<br>Divorced<br>Separated<br>Deserted<br>Never married	1<br>2<br>3<br>4<br>5<br>6<br>7	 →25<br>→25<br>→25<br>→25<br>→25<br>→25
(17)	How old was your husband at his last birthday?	Age in completed years	_ _	
(18)	Has your husband ever attended school?	Yes<br>No	1<br>2	 →20
(19)	What was the highest standard or degree / diploma he completed?<br><br>RECORD THE RESPONSE AS THE NUMBER OF YEARS OF EDUCATION COMPLETED	Completed years of education	_ _	
(20)	Is your husband currently employed?	Yes<br>No	1<br>2	
(21)	Has your husband been employed for at least a total period of 3 months in the previous 12 months?	Yes<br>No	1<br>2	 →23

(22)	What is his principal occupation, that is, what kind of work does he mainly do?<br><br>RECORD ONLY ONE OPTION. IF A RESPONDENT REPORTS MULTIPLE OCCUPATIONS, RECORD THE OCCUPATION WHERE HER HUSBAND SPENDS RELATIVELY MORE WORKING TIME.	Cultivators<br>Agricultural laborer<br>Worker in household industry<br>Other worker<br>Student<br>Household duties<br>Dependent<br>Pensioner<br>Rentier<br>Beggar<br>Other ______________________<br>(Specify)<br>No answer/do not want to answer	01<br>02<br>03<br>04<br>05<br>06<br>07<br>08<br>09<br>10<br>11<br><br>99	
(23)	Does your husband own this or any other house either alone or jointly with someone else?	Alone only<br>Jointly only<br>Both alone and jointly<br>Does not own	1<br>2<br>3<br>4	
(24)	Does your husband own any land either alone or jointly with someone else?	Alone only<br>Jointly only<br>Both alone and jointly<br>Does not own	1<br>2<br>3<br>4	
(25)	What is the religion of the head of household?	Hindu<br>Muslim<br>Christian<br>Sikh<br>Buddhist/Neo-Buddhist<br>Jain<br>Jewish<br>Parsi/Zoroastrian<br>No religion<br>Other ______________________<br>(Specify)	01<br>02<br>03<br>04<br>05<br>06<br>07<br>08<br>09<br>96	
(26)	What is the caste or tribe of the head of household?	Caste ______________________<br>(Specify)<br>Tribe ______________________<br>(Specify)<br>No caste/tribe<br>Don't know	991<br><br>992<br><br>993<br>998	 <br><br><br>→28
(27)	Is this a scheduled caste, a scheduled tribe, other backward class, or none of them?	Scheduled caste (SC)<br>Scheduled tribe (ST)<br>Other backward class (OBC)<br>None of them<br>Don't know	1<br>2<br>3<br>4<br>8	
(28)	How many people usually live in your household?	Current household size	_ _	
(29)	How many of these are children under the age of five?	Current number of children under-five	_ _	

(30)	Does anyone else other than your husband and your own children live in this household?	No (Nuclear)<br>Yes (Joint)	1<br>2	→32
(31)	SAY: I will now read to you the latest list of household members.<br><br>READ THE LIST OF HOUSEHOLD MEMBERS FOR THIS MOTHER FROM YOUR LAPTOP<br><br>ASK: Is this list correct or incorrect?<br><br>NOTE: IF YOU ARE UNABLE TO ACCESS YOUR LAPTOP, SKIP THIS QUESTION AND COMPLETE IT LATER	Correct<br>Incorrect	1<br>2	
(32)	Does any servant or maid usually live in this household?	Yes<br>No	1<br>2	

#	(33)<br>Please give me the names of all of your children, starting with the oldest.	(34)<br>Is [NAME] child male or female?	(35)<br>How old is the [NAME]?<br><br>RECORD AGE IN COMPLETED YEARS.	(36)<br>What is [NAME]'s date of birth?<br><br>ENTER '98' FOR DAY IF THE INFORMATION IS NOT AVAILABLE	(37)<br>Does [NAME] currently live in this household?
1		M / F	__	DD / MM / YYYY<br>_ _ / _ _ / _ _ _ _	Y / N
2		M / F	__	DD / MM / YYYY<br>_ _ / _ _ / _ _ _ _	Y / N
3		M / F	__	DD / MM / YYYY<br>_ _ / _ _ / _ _ _ _	Y / N
4		M / F	__	DD / MM / YYYY<br>_ _ / _ _ / _ _ _ _	Y / N
5		M / F	__	DD / MM / YYYY<br>_ _ / _ _ / _ _ _ _	Y / N

Pregnancy Status

No.	Questions and Filters	Coding Categories		SKIP
(38)	Are you pregnant now?	Yes<br>No<br>Unsure / don't know	1<br>2<br>8	 →40<br>→40
(39)	How many months pregnant are you?	Completed months	_ _	

No.	Questions and Filters	Coding Categories		SKIP
(40)	What is the main source of drinking water for members of your household?<br><br>RECORD ONE RESPONSE.	**Piped water**		
		Piped into dwelling	11	
		Piped to yard/plot	12	
		Public tap/standpipe	13	
		Tube well or borehole	21	
		**Dug well**		
		Protected well	31	
		Unprotected well	32	
		**Water from spring**		
		Protected spring	41	
		Unprotected spring	42	
		Rainwater	51	
		Tanker truck	61	
		Cart with small tank	71	
		Surface water (River/dam/ lake/pond/stream/canal/ irrigation channel)	81	
		Bottled water	91	
		Community RO plant	92	
		Other ______________________ (Specify)	96	
(41)	What kind of a toilet facility do members of your household usually use?<br><br>RECORD ONE RESPONSE.	**Flush or pour toilet**		
		Flush to piped sewer system	11	
		Flush to septic tank	12	
		Flush to pit latrine	13	
		Flush to somewhere else	14	
		Flush, don't know where	15	
		**Pit latrine**		
		Ventilated improved pit (VIP)/biogas latrine	21	
		Pit latrine with slab	22	
		Pit latrine without slab/open pit	23	
		Twin pit/composting toilet	31	
		Dry toilet	41	
		No facility/uses open space or field	51	→43
		Other ______________________ (Specify)	96	
(42)	Do you share this toilet with other households?	Yes	1	
		No	2	

No.	Questions and Filters	Coding Categories	Y	N	SKIP
(43)	Does your household have:		Y	N	
	a) Electricity?	Electricity	1	2	
	b) A mattress?	Mattress	1	2	
	c) A pressure cooker?	Pressure cooker	1	2	
	d) A chair?	Chair	1	2	
	e) A cot or bed?	Cot/bed	1	2	
	f) A table?	Table	1	2	
	g) An electric fan?	Electric fan	1	2	

	h) A radio or transistor?	Radio/transistor	1 2	
	i) A black and white television?	B & W television	1 2	
	j) A colour television	Colour television	1 2	
	k) A sewing machine?	Sewing machine	1 2	
	l) A mobile telephone?	Mobile telephone	1 2	
	m) A land line telephone?	Land line telephone	1 2	
	n) Internet?	Internet	1 2	
	o) A computer?	Computer	1 2	
	p) A refrigerator?	Refrigerator	1 2	
	q) An air conditioner/cooler?	Air conditioner/cooler	1 2	
	r) A washing machine?	Washing machine	1 2	
	s) A watch or clock?	Watch/clock	1 2	
	t) A bicycle?	Bicycle	1 2	
	u) A motorcycle or scooter?	Motorcycle/scooter	1 2	
	v) An animal-drawn cart?	Animal-drawn cart	1 2	
	w) A car?	Car	1 2	
	x) A water pump?	Water pump	1 2	
	y) A thresher?	Thresher	1 2	
	z) A tractor?	Tractor	1 2	
(44)	What type of fuel does your household mainly use for cooking?<br><br>RECORD ONE RESPONSE.	Electricity	01	
		LPG/natural gas	02	
		Biogas	03	
		Kerosene	04	
		Coal/lignite	05	
		Charcoal	06	
		Wood	07	
		Straw/shrubs/grass	08	
		Agricultural crop waste	09	
		Dung cakes	10	
		No food cooked in household	95	
		Other ______________________ (Specify)	96	
(45)	MAIN MATERIAL OF THE FLOOR<br><br>RECORD OBSERVATION.<br>RECORD ONE RESPONSE.	**Natural floor**		
		Mud/clay/earth	11	
		Sand	12	
		Dung	13	
		**Rudimentary floors**		
		Raw wood plans	21	
		Palm/bamboo	22	
		Brick	23	
		Stone	24	
		**Finished floor**		
		Parquet or polished wood	31	
		Vinyl or asphalt	32	
		Ceramic tiles	33	
		Cement	34	
		Carpet	35	
		Polished stone/marble/granite		
		Other ______________________	36	
		(Specify)	96	

(46)	MAIN MATERIAL OF THE ROOF<br><br>RECORD OBSERVATION.<br>RECORD ONE RESPONSE.	**Natural roofing**<br>No roof<br>Thatch/palm leaf/reed/grass<br>Mud<br>Sod/mud and grass mixture<br>Plastic/polythene sheeting<br>**Rudimentary roofing**<br>Rustic mat<br>Palm/bamboo<br>Raw wood planks/timber<br>Unburnt brick<br>Loosely packed stone<br>**Finished roofing**<br>Metal/GI<br>Wood<br>Calamine/cement fiber<br>Asbestos sheets<br>RCC/RBC/cement/concrete<br>Roofing shingles<br>Tiles<br>Slate<br>Burnt brick<br>Other ______________________<br>(Specify)	 11<br>12<br>13<br>14<br>15<br><br>21<br>22<br>23<br>24<br>25<br><br>31<br>32<br>33<br>34<br>35<br>36<br>37<br>38<br>39<br>96	
(47)	MAIN MATERIAL OF THE EXTERIOR WALLS<br><br>RECORD OBSERVATION.<br>RECORD ONE RESPONSE ONLY.	**Natural walls**<br>No walls<br>Cane/palm/trunks/bamboo<br>Mud<br>Grass/reeds/thatch<br>**Rudimentary walls**<br>Bamboo with mud<br>Stone with mud<br>Plywood<br>Cardboard<br>Unburnt brick<br>Raw wood/reused wood<br>**Finished walls**<br>Cement/concrete<br>Stone with lime/cement<br>Burnt bricks<br>Cement blocks<br>Wood planks/shingles<br>GI/metal/asbestos sheets<br>Other ______________________<br>(Specify)	 11<br>12<br>13<br>14<br><br>21<br>22<br>23<br>24<br>25<br>26<br><br>31<br>32<br>33<br>34<br>35<br>36<br>96	
(48)	How many rooms in this household are used for sleeping?	Rooms	_ _	
(49)	Does any member of this household own this household or any other house?	Yes<br>No	1<br>2	

(50)	Does any member of this household own any agricultural land?	Yes<br>No	1<br>2	 →52
(51)	How much agricultural land do members of this household own?<br><br>____________<br>(IF NOT IN ACRES, SPECIFY SIZE AND UNIT)	Acres	_ _ _ . _	
(52)	Does this household own any of the following animals:<br>a) Cows, bulls, or buffaloes?<br>b) Camels?<br>c) Horses, donkeys, or mules?<br>d) Goats?<br>e) Sheep?<br>f) Chickens or ducks?	 Cows/bulls/buffaloes<br>Camels<br>Horses/donkeys/mules<br>Goats<br>Sheep<br>Chickens/ducks	Y N<br>1 2<br>1 2<br>1 2<br>1 2<br>1 2<br>1 2	
(53)	Does any usual member of this household have a bank account or a post office account?	Yes<br>No<br>Don't know	1<br>2<br>8	
(54)	Is any usual member of this household covered by a health scheme or health insurance?	Yes<br>No<br>Don't know	1<br>2<br>8	 →57<br>→57
(55)	What type of health scheme or health insurance?<br>Any other type?<br><br>RECORD ALL MENTIONED	Employee state insurance scheme (ESIS)<br>Central government health scheme (CGHS)<br>State health insurance scheme<br>Rashtriya Swasthya Bima Yojana (RSBY)<br>Communite health insurance programme<br>Other health insurance through employer<br>Medical reimbursement from employer<br>Other privately purchased health insurance<br><br>Other ____________<br>(Specify)	A<br>B<br>C<br>D<br>E<br>F<br>G<br>H<br><br>X	
(56)	With what organization or company is your health scheme or health insurance?<br><br>IF THE RESPONDENT DOES NOT KNOW OR IS UNSURE, RECORD "DON'T KNOW/UNSURE".	____________<br>(Name(s) of insurance organization or company)		

(57)	When a child in your household gets sick, where do you generally take him/her for treatment?	______ (Name of provider)<br>______ (Name of facility/place)<br>______ (Name of village)	
(58)	What is the usual mode of transportation you use when you visit this provider?	Government ambulance 11<br>Other ambulance 12<br>Own jeep/car 21<br>Hired jeep/car (Taxi) 22<br>Own motorcycle/scooter 31<br>Hired motorcycle/scooter 32<br>Bus/train 41<br>Tempo/auto/tractor 51<br>Cart 61<br>On foot 71<br>Bicycle 81<br>Other ______ (Specify) 96	
(59)	When a child in your household has a serious illness, where do you generally take him/her for treatment?	______ (Name of provider)<br>______ (Name of facility/place)<br>______ (Name of village)	
(60)	What is the usual mode of transportation you use when you visit this provider?	Government ambulance 11<br>Other ambulance 12<br>Own jeep/car 21<br>Hired jeep/car (Taxi) 22<br>Own motorcycle/scooter 31<br>Hired motorcycle/scooter 32<br>Bus/train 41<br>Tempo/auto/tractor 51<br>Cart 61<br>On foot 71<br>Bicycle 81<br>Other ______ (Specify) 96	

(61)	Aside from the places that you have just mentioned, where else might you seek treatment or advice?<br>Anywhere else?<br><br>RECORD ALL FACILITIES AND PLACES MENTIONED. IF RESPONDENT MENTIONS NO OTHER PLACES, RECORD "NONE".	(Provider)<br>(Facility/place)<br>(Village)	(Provider)<br>(Facility/place)<br>(Village)	(Provider)<br>(Facility/place)<br>(Village)	
(62)	With whom would you usually discuss when deciding whether or not to seek care for your sick child?<br>Anyone else?<br><br>RECORD ALL MEMBERS REPORTED USING THEIR RELATIONSHIP TO RESPONDENT (FOR EXAMPLE: HUSBAND, FRIEND, SISTER, NEIGHBOR ETC.)	(Person(s) with whom respondent discusses care seeking listed in relationship to respondent)			
(63)	Check 43m: Does household own a mobile phone?	YES<br>NO			 →68
(64)	How many mobile phones does this household own?	Number of phones _ _			
(65)	Which members of your household primarily use these phones?<br><br>RECORD ALL MEMBERS REPORTED USING THEIR RELATIONSHIP TO RESPONDENT (FOR EXAMPLE: SELF, HUSBAND, MOTHER-IN-LAW, ETC.)	(Phones users listed in relationship to respondent)			
(66)	Are any of these phones smartphones?<br><br>Probe: Are you able to use or install applications on any of your phones, like WhatsApp or Facebook?	Yes 1<br>No 2			

(67)	For what purposes do you typically use your phone? Anything else?<br><br>RECORD ALL RESPONSES	Calling<br>Texting<br>Browsing internet<br>Playing games<br>Music<br>Movies<br>Photos<br>Other ______________________________<br>(Specify)	1<br>2<br>3<br>4<br>5<br>6<br>7<br>8	
(68)	Check: Is the mother in the phone group (study group ID = 1) or a comparison group (study group ID = 2-8)?<br>Phone group<br>Comparison Group			→69<br>→END

For questions 69-78, read the statement to the respondent and then read each response. Indicate the response by blackening the circle for the appropriate column.

#	Statement	Strongly disagree	Disagree	Neither agree nor disagree	Agree	Strongly Agree
(69)	I worry about charging the phone every day.	❍	❍	❍	❍	❍
(70)	I worry about keeping the phone on during the day	❍	❍	❍	❍	❍
(71)	I worry about carrying the phone with me when I seek treatment or advice for my sick child	❍	❍	❍	❍	❍
(72)	I worry that someone may try to steal the phone.	❍	❍	❍	❍	❍
(73)	I worry that the phone will not be easy to carry.	❍	❍	❍	❍	❍
(74)	I worry that I will lose the phone.	❍	❍	❍	❍	❍
(75)	I worry that I or someone in my household will damage the phone.	❍	❍	❍	❍	❍
(76)	I worry about my safety when I carry the phone.	❍	❍	❍	❍	❍
(77)	I worry that another family member will take the phone for himself or herself.	❍	❍	❍	❍	❍
(78)	Overall, I feel the phone will be easy to use.	❍	❍	❍	❍	❍

(79)	Do you plan to use the study phone as your primary phone?	Yes<br>No<br>Don't know/unsure	1<br>2<br>3
Ending time of the interview: ☐☐:☐☐			

Investigating Validity of Maternal Recall of Care-seeking Location
Collaborators: *Vadu Health and Demographic Surveillance System, Vadu Rural Health Program, KEM Hospital Research Centre Pune and University of Edinburgh, United Kingdom*

From 2: Follow-up Questionnaire for Care Seeking

Fill in one form per child

Starting time of the interview: □□:□□	Date: □□/□□/□□□□	Visit Number: □
Interviewer Name:	Mother's Name:	
Household Number (Record the HH No. Where the mother belongs):□□□ / □□□ / □□□□<br>Village Wadi Household Number		

Study ID Number: □□□	Study Group ID: □<br>(See codes below)	Phone ID Number: □□□<br>(Enter 998 if N/A)	Child ID Number: □

Codes for Study Group ID:

Phone Group	1
Longitudinal comparison group (visit at each follow-up)	2
Cross-sectional comparison group 1 (visit once at follow-up 1)	3
Cross-sectional comparison group 2 (visit once at follow-up 2)	4
Cross-sectional comparison group 3 (visit once at follow-up 3)	5
Cross-sectional comparison group 4 (visit once at follow-up 4)	6
Cross-sectional comparison group 5 (visit once at follow-up 5)	7
Cross-sectional comparison group 6 (visit once at follow-up 6)	8

Care seeking for childhood illness

No.	Questions and Filters	Coding Categories		SKIP
(1)	Has (NAME) had diarrhoea in the last 2 weeks?	YES<br>NO<br>DON'T KNOW	1<br>2<br>8	 →14<br>→14
(2)	Was there any blood in the stools?	YES<br>NO<br>DON'T KNOW	1<br>2<br>8	
(3)	Now I would like to know how much (NAME) was given to drink (including breastmilk) during the diarrhoea. Was (he/she) given less than usual to drink, about the same amount, or more than usual to drink?<br>IF LESS, PROBE: Was (he/she) given much less than usual to drink or somewhat less?	MUCH LESS<br>SOMEWHAT LESS<br>ABOUT THE SAME<br>MORE<br>NOTHING TO DRINK<br>DON'T KNOW	1<br>2<br>3<br>4<br>5<br>8	
(4)	When (NAME) had diarrhoea, was (he/she) given less than usual to eat, about the same amount, more than usual, or nothing to eat?<br>IF LESS, PROBE: Was (he/she) given much less than usual to eat or somewhat less?	MUCH LESS<br>SOMEWHAT LESS<br>ABOUT THE SAME<br>MORE<br>STOPPED FOOD<br>NEVER GAVE FOOD<br>DON'T KNOW	1<br>2<br>3<br>4<br>5<br>6<br>8	
(5)	Did you seek advice or treatment for the diarrhoea from any source?	YES<br>NO	1<br>2	 →10
(6)	Where did you seek advice or treatment?<br>Anywhere else?<br><br>RECORD ALL SOURCES MENTIONED	**PUBLIC HEALTH SECTOR**<br>GOVT./MUNICIPAL<br>HOSPITAL<br>VAIDYA/HAKIM/HOMEOPATH<br>(AYUSH)<br>GOVT. DISPENSARY	 <br>A<br><br>B<br>C	

	IF UNABLE TO DETERMINE IF A HOSPITAL, HEALTH CENTRE OR CLINIC IS PUBLIC OR PRIVATE HEALTH SECTOR, WRITE THE NAME OF THE PLACE(S).<br><br>____________________<br>(NAME OF FACILITY/PLACE(S))	UHC/UHP/UFWC D<br>CHC/RURAL HOSPITAL/ BLOCK PHC E<br>PHC/ADDITIONAL PHC F<br>SUB-CENTRE/ANM G<br>GOVT. MOBILE CLINIC H<br>CAMP I<br>ANGANWADI/ICDS CENTRE J<br>ASHA K<br>OTHER PUBLIC SECTOR HEALTH FACILITY L<br><br>NGO OR TRUST HOSPITAL/CLINIC M<br><br>**PRIVATE HEALTH SECTOR**<br>PVT. HOSPITAL N<br>PVT. DOCTOR/CLINIC O<br>PVT. PARAMEDIC P<br>VAIDYA/HAKIM/HOMEOPATH (AYUSH) Q<br>PHARMACY/DRUGSTORE R<br>OTHER PRIVATE SECTOR HEALTH FACILITY S<br><br>**OTHER SOURCE**<br>SHOP T<br>TRADITIONAL HEALER U<br>FRIEND/RELATIVE V<br><br>OTHER ____________ X<br>(SPECIFY)	
(7)	CHECK 6:	TWO OR MORE CODES SELECTED<br>ONLY ONE CODE SELECTED	→8<br>→9
(8)	Where did you first seek advice or treatment?<br><br>USE LETTER CODE FROM 6	FIRST PLACE __	
(9)	How many days after the diarrhoea began did you first seek advice or treatment for (NAME)?<br>IF THE SAME DAY, RECORD '00'.	DAYS _ _	
(10)	Was (he/she) given any of the following to drink at any time since (he/she) started having the diarrhoea:<br><br>a) A fluid made from a special packet called (LOCAL NAME FOR ORS PACKET)?<br><br>b) Gruel made from rice (OR OTHER LOCAL GRAIN)?	Y N DK<br><br>FLUID FROM ORS PKT 1 2 8<br><br>GRUEL 1 2 8	
(11)	Was (he/she) given zinc at any time since (he/she) started having diarrhoea?	YES 1<br>NO 2<br>DON'T KNOW 8	
(12)	Was anything (else) given to treat the diarrhoea?	YES 1<br>NO 2<br>DON'T KNOW 8	 →14<br>→14

(13)	What (else) was given to treat the diarrhoea? Anything else?<br><br>RECORD ALL TREATMENTS GIVEN.	**PILL OR ANTIBIOTIC**<br>ANTIBIOTIC<br>ANTIMOTILITY<br>OTHER (NON ANTIBIOTIC, ANTIMOTILITY, OR ZINC)<br>UKNOWN PILL OR SYRUP<br><br>**INJECTION**<br>ANTIBIOTIC<br>NON-ANTIBIOTIC<br>UNKNOWN INJECTION<br><br>INTRAVENOUS (IV)<br>HOME REMEDY/HERBAL MEDICINE<br><br>OTHER ______________________<br>(SPECIFY)	 A<br>B<br>C<br>D<br><br><br>E<br>F<br>G<br><br>H<br>I<br><br>X	
(14)	Has (NAME) been ill with a fever at any time in the last 2 weeks?	YES<br>NO<br>DON'T KNOW	1<br>2<br>8	 →16<br>→16
(15)	At any time during illness, did (NAME) have blood taken from (his/her) finger or heel for testing?	YES<br>NO<br>DON'T KNOW	1<br>2<br>8	
(16)	Has (NAME) had an illness with a cough at any time in the last 2 weeks?	YES<br>NO<br>DON'T KNOW	1<br>2<br>8	 →19<br>→19
(17)	When (NAME) had an illness with a cough, did (he/she) breathe faster than usual with short, rapid breaths or have difficulty breathing?	YES<br>NO<br>DON'T KNOW	1<br>2<br>8	 →20<br>→20
(18)	Was the fast or difficult breathing due to a problem in the chest or to a blocked or runny nose?	CHEST ONLY<br>NOSE ONLY<br>BOTH<br>OTHER ______________________<br>(SPECIFY)<br><br>DON'T KNOW	1<br>2<br>3<br>6<br><br><br>8	→20<br>→20<br>→20<br>→20<br><br><br>→20
(19)	CHECK 14: HAD FEVER		YES<br>NO OR DON'T KNOW	→20<br>→33
(20)	Now I would like to know how much (NAME) was given to drink (including breastmilk) during the illness with a (fever/cough). Was (he/she) given less than usual to drink, about the same amount, or more than usual to drink?<br>IF LESS, PROBE: Was (he/she) given much less than usual to drink or somewhat less?	MUCH LESS<br>SOMEWHAT LESS<br>ABOUT THE SAME<br>MORE<br>NOTHING TO DRINK<br>DON'T KNOW	1<br>2<br>3<br>4<br>5<br>8	
(21)	When (NAME) had a (fever/cough), was (he/she) given less than usual to eat, about the same amount, more than usual, or nothing to eat?<br>IF LESS, PROBE: Was (he/she) given much less than usual to eat or somewhat less?	MUCH LESS<br>SOMEWHAT LESS<br>ABOUT THE SAME<br>MORE<br>STOPPED FOOD<br>NEVER GAVE FOOD<br>DON'T KNOW	1<br>2<br>3<br>4<br>5<br>6<br>8	

(22)	Did you seek advice or treatment for the illness from any source?	YES<br>NO	1<br>2	 →27
(23)	Where did you seek advice or treatment?<br>Anywhere else?<br><br>RECORD ALL SOURCES MENTIONED<br><br>IF UNABLE TO DETERMINE IF A HOSPITAL, HEALTH CENTRE OR CLINIC IS PUBLIC OR PRIVATE HEALTH SECTOR, WRITE THE NAME OF THE PLACE(S).<br><br>____________________<br>(NAME OF FACILITY/PLACE(S))	**PUBLIC HEALTH SECTOR**<br>GOVT./MUNICIPAL HOSPITAL<br>VAIDYA/HAKIM/HOMEOPATH (AYUSH)<br>GOVT. DISPENSARY<br>UHC/UHP/UFWC<br>CHC/RURAL HOSPITAL/ BLOCK PHC<br>PHC/ADDITIONAL PHC<br>SUB-CENTRE/ANM<br>GOVT. MOBILE CLINIC<br>CAMP<br>ANGANWADI/ICDS CENTRE<br>ASHA<br>OTHER PUBLIC SECTOR HEALTH FACILITY<br><br>NGO OR TRUST HOSPITAL/CLINIC<br><br>**PRIVATE HEALTH SECTOR**<br>PVT. HOSPITAL<br>PVT. DOCTOR/CLINIC<br>PVT. PARAMEDIC<br>VAIDYA/HAKIM/HOMEOPATH (AYUSH)<br>PHARMACY/DRUGSTORE<br>OTHER PRIVATE SECTOR HEALTH FACILITY<br><br>**OTHER SOURCE**<br>SHOP<br>TRADITIONAL HEALER<br>FRIEND/RELATIVE<br><br>OTHER ________________<br>(SPECIFY)	 A<br>B<br>C<br>D<br>E<br>F<br>G<br>H<br>I<br>J<br>K<br>L<br><br>M<br><br><br>N<br>O<br>P<br>Q<br>R<br>S<br><br><br>T<br>U<br>V<br><br>X	
(24)	CHECK 23:	TWO OR MORE CODES SELECTED<br>ONLY ONE CODE SELECTED		→25<br>→26
(25)	Where did you first seek advice or treatment?<br><br>USE LETTER CODE FROM 23.	FIRST PLACE	__	
(26)	How many days after the illness began did you first seek advice or treatment for (NAME)?<br><br>IF THE SAME DAY, RECORD '00'.	DAYS	_ _	
(27)	At any time during the illness, did (NAME) take any drugs for the illness?	YES<br>NO<br>DON'T KNOW	1<br>2<br>8	 →31<br>→31
(28)	What drugs did (NAME) take?<br>Any other drugs?	**ANTIMALARIAL DRUGS**<br>CHLOROQUINE<br>PRIMAQUINE	 A<br>B	

	RECORD ALL TREATMENTS GIVEN	SP/FANSIDAR<br>QUININE<br>ARTEMISININ COMBINATION THERAPY<br>OTHER ANTIMALARIAL<br>UNKNOWN ANTIMALARIAL<br><br>ANTIBIOTIC DRUG<br><br>**OTHER DRUGS**<br>ASPIRIN<br>ACETAMINOPHIN<br>IBUPROFEN<br><br>OTHER ______________________<br>(SPECIFY)<br><br>UNKNOWN DRUG	C<br>D<br>E<br>F<br>G<br><br>H<br><br><br>I<br>J<br>K<br><br>X<br><br><br>Z	
(29)	CHECK 28: ANY CODE ‘A-G’ RECORDED		YES<br>NO	→30<br>→31
(30)	How long after the fever started, did (NAME) first take (DRUG(S) FROM 28 A-G)?	SAME DAY<br>NEXT DAY<br>TWO DAYS AFTER FEVER<br>THREE OR MORE DAYS AFTER FEVER<br>DON’T KNOW	1<br>2<br>3<br>4<br>8	
(31)	Are you pregnant now?	Yes<br>No<br>Unsure / don’t know	1<br>2<br>8	 →33<br>→33
(32)	How many months pregnant are you?<br><br>RECORD NUMBER OF COMPLETED MONTHS.	Completed months	_ _	
(33)	CHECK 1, 14, AND 16: ANY CODE ‘1’ (‘YES’) RECORDED		YES<br>NO	→34<br>→END

Presence and timing of symptoms

Symptoms	(A)<br>In the previous two weeks, was [NAME] ill with [SYMPTOM]?<br><br>IF NO, SKIP TO NEXT SYMPTOM.	(B)<br>How long ago did [SYMPTOM] begin?<br><br>RECORD RESPONSE IN DAYS.	(C)<br>Is [NAME] currently ill with [SYMPTOM]?<br><br>IF "YES" SKIP TO NEXT SYMPTOM.	(D)<br>If "No:" ask: How many days ago did [SYMPTOM] end?<br><br>RECORD AS DAYS. RECORD, "1" FOR YESTERDAY, "2" FOR THE DAY BEFORE, ETC.	(E)<br>(ONLY ASK IF RESPONDENT REPORTED CONVULSIONS IN PREVIOUS TWO WEEKS)<br>How many times did convulsions occur as part of this illness?
(34) Diarrhea	Yes 1<br>No 2<br>Don't know 8	Days _ _ _	Yes 1<br>No 2	Days _ _	
(35) Blood in stools	Yes 1<br>No 2<br>Don't know 8<br>Skipped 9	Days _ _ _	Yes 1<br>No 2	Days _ _	
(36) Fever	Yes 1<br>No 2<br>Don't know 8	Days _ _ _	Yes 1<br>No 2	Days _ _	
(37) Cough	Yes 1<br>No 2<br>Don't know 8	Days _ _ _	Yes 1<br>No 2	Days _ _	
(38) Fast breathing	Yes 1<br>No 2<br>Don't know 8<br>Skipped 9	Days _ _ _	Yes 1<br>No 2	Days _ _	
(39) Unable to eat	Yes 1<br>No 2<br>Don't know 8	Days _ _ _	Yes 1<br>No 2	Days _ _	
(40) Vomited everything	Yes 1<br>No 2<br>Don't know 8	Days _ _ _	Yes 1<br>No 2	Days _ _	
(41) Convulsions	Yes 1<br>No 2<br>Don't know 8	Days _ _ _	Yes 1<br>No 2	Days _ _	Occurrences _ _
(42) Unusually sleepy or unconscious	Yes 1<br>No 2<br>Don't know 8	Days _ _ _	Yes 1<br>No 2	Days _ _	
(43) Lower chest indrawing	Yes 1<br>No 2<br>Don't know 8	Days _ _ _	Yes 1<br>No 2	Days _ _	
(44) Nasal discharge ("runny nose")	Yes 1<br>No 2<br>Don't know 8	Days _ _ _	Yes 1<br>No 2	Days _ _	
(45) Other problem (specify):<br>______	Yes 1<br>No 2<br>Don't know 8	Days _ _ _	Yes 1<br>No 2	Days _ _	
(46)	How serious was this illness?			Very serious 1<br>Somewhat serious 2<br>Not serious 3	

Investigating Validity of Maternal Recall of Care-seeking Location
Collaborators: *Vadu Health and Demographic Surveillance System, Vadu Rural Health Program, KEM Hospital Research Centre Pune and University of Edinburgh, United Kingdom*

Treatment within and outside the household

(47)	In the previous two weeks, did you avail of any treatment either at home or outside for the illness?<br>Yes 1<br>No 2	→What was the first thing you did? (Fill Row 1)<br>→End questionnaire

(48) Seq ID	(49) Within/ outside of family	Type of action			(52) How many days ago did this take place? RECORD THE RESPONSE IN DAYS. RECORD 0 IF THE ACTION WAS TAKEN TODAY, 1 FOR YESTERDAY, ETC.	(53) Did [NAME] stay overnight at [FACILITY] for treatment?	(54) How many nights did [NAME] stay at [FACILITY] for treatment?	***ASK THESE TWO QUESTIONS TO PHONE GROUP ONLY***		(57) Did you do anything after this for the illness either within or outside the family? IF YES, PROCEED TO FILL IN THE NEXT ROW. IF "NO" CONTINUE TO QUESTION (56).
		(50) What did you do? RECORD CODE FROM NEXT PAGE. IF CODE A01-A06, ASK 52 AND 57 ONLY.(SKIP 51, 53-56)		(51) What is the name of the facility that you visited?				(55) Did you carry the study phone when you visited [PROVIDER]?	(56) If "No" ask: "Why did you not carry your phone?" RECORD ALL THAT APPLY USING CODES FROM NEXT PAGE.	
		CODE	IF "OTHER", SPECIFY:							
1	IN / OUT	_ _ _			_ _	Y / N	_ _	Y / N	A B C D E<br>F: ____________	Y / N
2	IN / OUT	_ _ _			_ _	Y / N	_ _	Y / N	A B C D E<br>F: ____________	Y / N
3	IN / OUT	_ _ _			_ _	Y / N	_ _	Y / N	A B C D E<br>F: ____________	Y / N
4	IN / OUT	_ _ _			_ _	Y / N	_ _	Y / N	A B C D E<br>F: ____________	Y / N
5	IN / OUT	_ _ _			_ _	Y / N	_ _	Y / N	A B C D E<br>F: ____________	Y / N
6	IN / OUT	_ _ _			_ _	Y / N	_ _	Y / N	A B C D E<br>F: ____________	Y / N
7	IN / OUT	_ _ _			_ _	Y / N	_ _	Y / N	A B C D E<br>F: ____________	Y / N

**Codes for Question (50): Type of treatment sought within/outside the home**				**Codes for Question (56): Reasons mother did not bring the phone**	
**Treatment within the home**		**Treatment outside the home**		Forgot	A
Apply heat	A01	*Public health sector*		Phone with husband	B
Herbs/herbal tea	A02	Govt./municipal hospital	B01	Phone with other person (not husband)	C
Change of diet	A03	Vaidya/hakim/homeopath (AYUSH)	B02	Phone not working	D
Oral rehydration solution or therapy (ORS/ORT)	A04	Govt. dispensary	B03	No battery charge	E
Used leftover pills/medicines	A05	UHC/UHP/UFWC	B04	Other (specify)	F
		CHC/Rural hospital/Block PHC	B05		
Other treatment within the home (specify)	A06	PHC/Additional PHC	B06		
		Sub-centre/ANM	B07		
		Govt. mobile clinic	B08		
		Camp	B09		
		Anganwadi/ICDS centre	B10		
		ASHA	B11		
		Other public health sector facility	B12		
		NGO or trust hospital/clinic	B13		
		Private health sector			
		Pvt.. hospital	B14		
		Pvt. doctor/clinic	B15		
		Pvt. paramedic	B16		
		Vaidya/hakim/homeopath (AYUSH)	B17		
		Pharmacy/drugstore	B18		
		Other private health sector facility	B19		
		Other source			
		Shop	B20		
		Traditional healer	B21		
		Other treatment outside the home (specify)	B22		

(58)	CHECK 49: DID THE RESPONDENT SEEK ANY CARE FROM **OUTSIDE** THE HOME?		
	Yes	1	→ CONTINUE TO 59
	No	2	→ SKIP TO 73

Say: **“**I would now like to ask you some information about the costs for treatment that you sought outside the home.”

(59) Enter Seq ID of Q48.	(60) Total Amount (Rs.)	(61) Consultation Fee Amount	Medicines				Diagnostics		Transport			Other Costs	
			(62) Medicines Amount (Rs.)	(63) Number of different medicines purchased	(64) Name of medicine purchased	(65) # Days Supply	(66) Diagnostics Amount (Rs.)	(67) Type of test (e.g. X-Ray)	(68) Trans. Amount (Rs.)	(69) Main Mode of Transport	(70) Total Time (Minutes; round-trip)	(71) Other Costs Amount (Rs.)	(72) What do these include?
					a.			a.					a.
					b.			b.					b.
_				□□	c.			c.					c.
					d.			d.					d.
					e.			e.					e.
					a.			a.					a.
					b.			b.					b.
_				□□	c.			c.					c.
					d.			d.					d.
					e.			e.					e.
					a.			a.					a.
					b.			b.					b.
_				□□	c.			c.					c.
					d.			d.					d.
					e.			e.					e.

Codes for Question (68)

11 Government ambulance
12 Other ambulance
21 Own jeep/car
22 Hired jeep/car
23 Shared Taxi / Autorickshaw
31 Own motorcycle/scooter
32 Hired motorcycle/scooter
41 Bus/train
51 Tempo/auto/tractor
61 Cart
71 On foot
81 Bicycle
96 Other (specify)

	Mother / primary caregiver	Accompanying person (if any)
(73) Total # of days lost due to caring for child's illness		
(74) Monthly earnings (record 0 if does not ear a wage)		

(75)	How were expenditures related to this illness managed?<br>For how much?<br><br>RECORD ALL THAT APPLY	Current income of any household member	A	Rs. _ _ _ _ _ _ _
		Savings (e.g. bank account)	B	Rs. _ _ _ _ _ _ _
		Payment or reimbursement from health insurance plan	C	Rs. _ _ _ _ _ _ _
		Sold assets (e.g. furniture, animals, jewelry)	D	Rs. _ _ _ _ _ _ _
		Borrowed from family members or friends from outside this household	E	Rs. _ _ _ _ _ _ _
		Borrowed from someone other than a family member or friend	F	Rs. _ _ _ _ _ _ _
		Pawning/hawking/mortgage/guarantee	G	Rs. _ _ _ _ _ _ _
		Other______________________<br>(Specify)	X	Rs. _ _ _ _ _ _ _
Ending time of the interview:□□				

Investigating Validity of Maternal Recall of Care-seeking Location
Collaborators: *Vadu Health and Demographic Surveillance System, Vadu Rural Health Program, KEM Hospital Research Centre Pune and University of Edinburgh, United Kingdom*

Form 3: Follow up Care-seeking Questions for Mothers in the Phone Group

Starting time of the interview: □□:□□	Date: □□/□□/□□□□	Interviewer Name:	Mother's Name:
HDSS Id: □□□/□□□/□□□□	Study Id Number: □□□	Phone Id: □□□	Which follow-up visit is this? (Record 1-9 for the month of visit) □	Pregnancy status (Copy from form 2, Q. 32) □

Sr. No.	Date	Provider			(1) Visit?	(2) Seek treatment or advice?	(3) Avail of treatment or advice?	(4) U5 and/or mother?	(5) For whom? (Circle all that apply)		(6) What was the purpose of [NAME]'s/[YOUR] visit to this provider? (Record all that apply using codes on next page.)				(7) Time at provider (minutes)	(8) Link ID	
		ID	Name	Village					U5 Child	M	Child 1	Child 2	Child 3	Mother		Link Sr. No.	Link Note
					Y 1<br>N 2<br>DK 3	Y 1<br>N 2<br>DK 3	Y 1<br>N 2<br>DK 3	Y 1<br>N 2<br>DK 3	1 2 3	4							
					Y 1<br>N 2<br>DK 3	Y 1<br>N 2<br>DK 3	Y 1<br>N 2<br>DK 3	Y 1<br>N 2<br>DK 3	1 2 3	4							
					Y 1<br>N 2<br>DK 3	Y 1<br>N 2<br>DK 3	Y 1<br>N 2<br>DK 3	Y 1<br>N 2<br>DK 3	1 2 3	4							
					Y 1<br>N 2<br>DK 3	Y 1<br>N 2<br>DK 3	Y 1<br>N 2<br>DK 3	Y 1<br>N 2<br>DK 3	1 2 3	4							
					Y 1<br>N 2<br>DK 3	Y 1<br>N 2<br>DK 3	Y 1<br>N 2<br>DK 3	Y 1<br>N 2<br>DK 3	1 2 3	4							
					Y 1<br>N 2<br>DK 3	Y 1<br>N 2<br>DK 3	Y 1<br>N 2<br>DK 3	Y 1<br>N 2<br>DK 3	1 2 3	4							

(Include additional rows as necessary)

Investigating Validity of Maternal Recall of Care-seeking Location
Collaborators: *Vadu Health and Demographic Surveillance System, Vadu Rural Health Program, KEM Hospital Research Centre Pune and University of Edinburgh, United Kingdom*

**Guide**	**Purposes for children:**	**Purposes for mother:**
(1) Visit: Did you visit the provider on this date? (Continue to Question 2 if "Yes". Otherwise, skip to the next row.)<br>(2) Seek medical care: Did you seek treatment or advice from this provider? (Continue to Question 3 if "Yes". Otherwise, skip to the next row.)<br>(3) Avail of medical care: Were you able to avail of treatment or advice at the provider?<br>(4) U5 and/or mother: Were any of those services related to your or your child's illness or related to your current/most recent pregnancy?<br>(Only ask the second half if the mother is currently pregnant. Continue to Question 4 if "Yes" to either. Otherwise, skip to the next row.)<br>(5) For whom: For whom did you seek treatment or advice? (Continue to "Purpose" for each circled individual)<br>(6) Purpose: What was the purpose of [NAME]'s visit to this provider? (Record all that apply using codes below.)<br>(7) Time at provider: Approximately how many minutes did you spend during this visit to [PROVIDER]?<br>(8) Link ID: Did you previously visit a provider for this same purpose? (If no, skip to next row. If yes, enter the serial number that corresponds to the provider that the respondent previously visited. If the respondent reports previous care seeking from a provider that is not listed on the form, enter "96" as the link serial number and write a note with information on the provider. The note should include whatever information the mother provides. For example, she may report the date and provider name.)	A1: Treatment for diarrhea<br>A2: Treatment for fever/cough<br>A3: Treatment for other condition<br>A4: Other service for child	B1: Antenatal care visit<br>B2: Delivery<br>B3: Checking mother's health post-delivery<br>B4: Checking baby's health post-delivery<br>B8: Other specify
	Ending time of the interview: □□:□□	

Investigating Validity of Maternal Recall of Care-seeking Location
Collaborators: *Vadu Health and Demographic Surveillance System, Vadu Rural Health Program, KEM Hospital Research Centre Pune and University of Edinburgh, United Kingdom*

Form 4. Healthcare Facility / Provider Questionnaire

Time of the observation: □□:□□			
Interviewer Name:	Signature:		Date: □□/□□/□□□□
Facility / Provider Name:			Provider ID: □□□
Village:		Address:	

(1)	GPS Device ID □□	(2)	Number of satellites in range	□□	
(3)	Accuracy □□	(4)	Elevation	□□□	
(5)	Latitude	North<br>□		Degree and decimal<br>□□.□□□□□□	
(6)	Longitude	East<br>□		Degree and decimal<br>□□.□□□□□□	
(7)	Way Point	01 In front of provider		02 Place near provider:	
(8)	What is the type of health provider?	**Public health sector**<br>Govt./municipal hospital<br>Vaidya/hakim/homeopath (AYUSH)<br>Govt. dispensary<br>UHC/UHP/UFWC<br>CHC/Rural hospital/Block PHC<br>PHC/Additional PHC<br>Sub-centre/ANM<br>Govt. mobile clinic<br>Camp<br>Anganwadi/ICDS centre<br>ASHA<br>Other public health sector facility<br><br>NGO or trust hospital/clinic<br><br>**Private health sector**<br>Pvt.. hospital<br>Pvt. doctor/clinic<br>Pvt. paramedic<br>Vaidya/hakim/homeopath (AYUSH)<br>Pharmacy/drugstore<br>Other private health sector facility<br><br>**Other source**<br>Shop<br>Traditional healer<br><br>Other ________________________<br>(SPECIFY)			 A<br>B<br>C<br>D<br>E<br>F<br>G<br>H<br>I<br>J<br>K<br>L<br><br>M<br><br><br>N<br>O<br>P<br>Q<br>R<br>S<br><br>T<br>U<br><br>X

Investigating Validity of Maternal Recall of Care-seeking Location
Collaborators: *Vadu Health and Demographic Surveillance System, Vadu Rural Health Program, KEM Hospital Research Centre Pune and University of Edinburgh, United Kingdom*

For each provider at this facility, collect the age, sex, highest completed training, and duration at this health facility.

#	(9) What is the provider age?	(10) What is the provider sex?	(11) What is the highest level completed by him/her in medical training? Code	If “X,” specify:	(12) How long has s/he worked in this health facility? RECORD RESPONSE IN COMPLETE YEARS
1	☐☐	M / F	☐		☐☐
2	☐☐	M / F	☐		☐☐
3	☐☐	M / F	☐		☐☐
4	☐☐	M / F	☐		☐☐
5	☐☐	M / F	☐		☐☐

Codes for Question 12:

BAMS ……………A	BHMS ……………E	D-Pharm…………..I
BNYS ……………B	MBBS ……………F	Other ……………X
BUMS ……………C	MD…..……………G	
BSMS ……………D	B-Pharm…….……H	

(13)	In what year did this facility start?	Year	☐☐☐☐
(14)	Does this facility provide patient treatment on an outpatient basis, an inpatient basis, both or neither?	Outpatient<br>Inpatient<br>Both<br>Neither	1<br>2<br>3<br>4
(15)	Does this facility dispense drugs, prescribe drugs, both dispense and prescribe drugs, or neither?	Dispense<br>Prescribe<br>Both<br>Neither	1<br>2<br>3<br>4
(16)	Does this facility perform diagnostic tests within at this location or do you refer to another facility for diagnostics?	At this location<br>Refer<br>Do not provide diagnostics	1<br>2<br>3
(17)	Does this facility usually offer services in allopathic medicine, Ayurveda, Unani, homeopathy, any other type of medicine, or some combination of these?<br><br>RECORD ALL THAT APPLY	Allopathic<br>Ayurveda<br>Unani<br>Homeopathy<br><br>Other ______________ (SPECIFY)	A<br>B<br>C<br>D<br><br>X
(18)	Does the provider currently offer any of the following services:<br><br>a) Family planning, including spacing methods<br>b) Immunization	 <br>Family planning<br><br>Immunization	Y N<br><br>1 2<br><br>1 2

	c) Antenatal care	Antenatal care	1	2
	d) Delivery care	Delivery care	1	2
	e) Cesarean section	C-Section	1	2
	f) Postnatal care	Postnatal care	1	2
	g) Disease prevention	Disease prevention	1	2
	h) Medical treatment for adults or children over 5 years of age	Medical treatment for adults	1	2
	i) Medical treatment for children under 5 years of age	Medical treatment for children	1	2
	j) Growth monitoring	Growth monitoring	1	2
	k) Routine health check-ups	Check-ups	1	2
	l) Distributing medicines	Distributing medicines	1	2
	x) Other services ________________ (Specify)	Other services	1	2
(19)	Do you usually have:		Y	N
	a) Any antibiotic?	Any antibiotic	1	2
	b) Oral rehydration salts (ORS)?	Oral rehydration salts	1	2
	c) Zinc sulfate?	Zinc sulfate	1	2
	d) Any antimalarial drug?	Any antimalarial drug	1	2
(20)	Do you usually offer:		Y	N
	a) X-Ray exam?	X-Ray	1	2
	b) Peripheral blood smear for malaria parasite?	Peripheral blood smear for malaria parasite	1	2
(21)	Do you charge for any services offered at the facility?	Yes<br>No	1<br>2	
(22)	Do you accept payment in installments for any of these services?	Yes<br>No	1<br>2	
(23)	Do you provide a cashless facility?	Yes<br>No<br>Don't know	1<br>2<br>3	 →25<br>→25
(24)	For which insurance companies do you offer a cashless facility?<br><br>RECORD EACH RESPONSE ON A SEPARATE LINE	a. ______<br>b. ______<br>c. ______<br>d. ______<br>e. ______<br>f. ______<br>g. ______<br>h. ______<br>i. ______		

For Question 25-32: Ask "When does this facility usually offers services on _________?" Replace the blank with the word next to the question. For example, "When does the staff of this health facility usually see patients on Monday?"

Record a response by making an "✓" in the appropriate column(s). Record all responses given. If the facility does not usually offer services on a specific day, put a "✓ in the column for "Not available."

		Morning	Afternoon	Evening	Night	Other	If Other, specify:	Not available
(26)	Monday							
(27)	Tuesday							
(28)	Wednesday							
(29)	Thursday							
(30)	Friday							
(31)	Saturday							
(32)	Sunday							
(33)	Holidays							

(34)	Does the provider stay in the same premises / same village as the facility where s/he provides care?	Yes<br>No	1<br>2
(35)	How much time does a provider typically take to attend to an emergency?	<30 minutes<br>30 minutes to an hour<br>1-3 hours<br>More than 3 hours	1<br>2<br>3<br>4

Ending time of the interview: ☐☐:☐☐

Investigating Validity of Maternal Recall of Care-seeking Location
Collaborators: *Vadu Health and Demographic Surveillance System, Vadu Rural Health Program, KEM Hospital Research Centre Pune and University of Edinburgh, United Kingdom*

Form 5: HOUSEHOLD VISIT FORM

Instructions: Administer this form to mothers in the phone group on Day-3 after study enrollment, once between Day-11 and Day-14 after study enrollment, and at each monthly follow-up visit

Part 1: IDENTIFYING INFORMATION

Starting time of the interview: □□:□□	Date: □□/□□/□□□□	
Interviewer Name:	Mother's Name:	
HDSS Id: □□□/□□□/□□□□	Study ID Number: □□□	Phone ID Number: □□□

Which follow-up visit is this? (Circle ONE letter?

Day 3……………………A
Day 11-14………………B
Month 1…………………C
Month 2…………………D
Month 3…………………E
Month 4…………………F
Month 5…………………G
Month 6…………………H
Other ____________ ...X
(Specify)

PART 2: QUESTIONNAIRE FOR MOTHER

For questions 1-4, read the responses to the respondent and mark the appropriate column.

		Never	□□□□□ □	Sometimes	Often	Always
1	Since the last visit, how often did you use the phone?	❍	❍	❍	❍	❍
2	Since the last visit, how often did you charge your phone?	❍	❍	❍	❍	❍
3	Since the last visit, how often did someone other than you carry the phone?	❍	❍	❍	❍	❍
4	Since the last visit, how often did you have problems with the phone not working?<br><br>IF 'NEVER', SKIP TO QUESTION 7.	❍	❍	❍	❍	❍
5	What were the problems?<br>____________________<br>____________________<br>____________________					

6	Since the last visit, how often were you were able to solve any problems with the phone?	Never<br>Occasionally<br>Frequently<br>Always	1<br>2<br>3<br>4

7	Since the last visit, what was the most common purpose for using the phone?<br><br>RECORD **<u>ONE</u>** RESPONSE	CALLS<br>SMS<br>INTERNET<br>CAMERA<br>GAMES<br>MUSIC<br>MOVIES<br>OTHER_______________________<br>(SPECIFY)	A<br>B<br>C<br>D<br>E<br>F<br>G<br>X	
8	Since the last visit, how else did you use the phone?<br><br>RECORD **<u>ALL</u>** RESPONSES	CALLS<br>SMS<br>INTERNET<br>CAMERA<br>GAMES<br>MUSIC<br>MOVIES<br>OTHER_______________________<br>(SPECIFY)	A<br>B<br>C<br>D<br>E<br>F<br>G<br>X	
9	Do you have any questions or comments about the phone?	Yes<br>No	1<br>2	 →11
10	What are your questions or comments?<br><br>___________________________________<br>___________________________________<br>___________________________________<br>___________________________________			

Investigating Validity of Maternal Recall of Care-seeking Location
Collaborators: *Vadu Health and Demographic Surveillance System, Vadu Rural Health Program, KEM Hospital Research Centre Pune and University of Edinburgh, United Kingdom*

PART 3: INTERVIEWER CHECKLIST

Begin this section by saying: “Now I would like to check a few things on the phone. May I see it?”

11	Is the phone available when you visit?	Yes 1<br>No 2	→13
12	Who currently has the phone?<br>____________________<br>(Record who has the phone using his/her relationship to the respondent. For example, friend, sister, husband, etc.)<br>END QUESTIONNAIRE.		
13	Is the phone turned on?<br>If not: remind the mother that the phone should remain turned on.	Yes 1<br>No 2	
14	Is the CareTrack application installed?<br>If not: remind the mother that the application should not be uninstalled.	Yes 1<br>No 2	→16
15	How many points are currently saved in the CareTrack application?	□□□□□	
16	Is Mobile Data turned on?<br>If not: remind the mother that mobile data should remain on.	Yes 1<br>No 2	
17	Is the data plan using the study SIM?<br>If not: remind the mother that the study SIM should be used for mobile data.	Yes 1<br>No 2	
18	Is the WiFi turned on?<br>If not: remind the mother that WiFi should always remain on.	Yes 1<br>No 2	
19	Are location services turned on?<br>If not: remind the mother not that location services should remain on.	Yes 1<br>No 2	
20	Is the location mode set to “High accuracy”?<br>If not: remind the mother that the location mode should remain set to “High accuracy”.	Yes 1<br>No 2	→22
21	What is the setting for the location mode?	Battery saving 1<br>Device only 2	
22	Is “STAMINA mode” turned off?<br>If not: remind the mother that STAMINA mode should remain off.	Yes 1<br>No 2	
23	What is the current percent battery charge?	□□□%	
24	Please write down any notes from your visit with the mother.		
Ending time of the interview: □□:□□			
